# SWIFT-Mamba: A Lightweight State Space Model Toward In Situ Verification of Airborne Wavefront Sensing Signals

**DOI:** 10.3390/s26165048

**Published:** 2026-08-09

**Authors:** Jianbao Ma, Hao Wang, Yiyou Fan, Wei Jiang, Jinshan Su

**Affiliations:** 1 Key Laboratory of Signal Intelligent Capture and New Generation Communication Technology, School of Electronic Engineering, Yili Normal University, 448 Jiefang Road, Yining 835000, China; 20240050184@ylnu.edu.cn (J.M.); 20250050227@ylnu.edu.cn (H.W.); 14012@ylnu.edu.cn (Y.F.); 2 Key Laboratory of Intelligent Optical Sensing and Manipulation, College of Engineering & Applied Sciences, Ministry of Education, Nanjing University, 163 Xianlin Ave, Nanjing 210023, China; weijiang@nju.edu.cn

**Keywords:** sub-millimeter vibration signal enhancement, Mamba, lightweight neural network, wavefront sensor, time-frequency analysis

## Abstract

Unmanned Aerial Vehicle (UAV)-borne laser wavefront sensing technology holds significant application prospects for high-precision vibration detection in the field; however, the acquired signals are highly susceptible to complex, nonlinear environmental noise interference. Existing high-precision deep learning denoising models typically rely on massive computational resources, making them difficult to deploy on resource-constrained edge devices. Consequently, practical engineering exploration is often confined to an inefficient “blind sampling followed by offline processing” mode, incurring a high risk of data invalidation. To explore solutions for real-time quality control at the edge, this paper proposes a lightweight time-frequency state space model (SWIFT-Mamba), aiming to provide an efficient algorithmic foundation and an engineering proof-of-concept for portable devices moving toward in situ verification. Through rigorous evaluation on over 60,000 laboratory-measured and controlled synthetic wavefront vibration data samples, SWIFT-Mamba achieves an average Signal-to-Noise Ratio (SNR) gain of 19.64 dB and a Scale-Invariant Signal-to-Distortion Ratio (SI-SDR) gain of 16.10 dB, with an extremely low computational overhead requiring only 0.066 M parameters and 0.147 GFLOPs. Experimental results demonstrate that while significantly reducing computational costs, the proposed model can effectively extract the physical manifold of the signal and precisely preserve high-frequency phase features.

## 1. Introduction

Laser remote sensing technology based on wavefront sensors, owing to its high phase sensitivity and non-contact measurement advantages, has demonstrated significant application potential in fields such as field micro-vibration detection, geological exploration, and structural health monitoring [1,2,3]. To clarify the fundamental physical context, the underlying quantity being measured is the highly transient, sub-millimeter-scale dynamic vertical displacement of a target surface excited by mechanical or seismic waves. When a continuous probe laser irradiates this vibrating surface, these minute physical displacements induce proportional spatial phase distortions in the reflected laser wavefront. A wavefront sensor utilizes a micro-lens array to capture these distortions, mapping them into the lateral geometric displacements of focal spots on the detector plane. The dynamic deviations of these spots from their static reference positions are continuously extracted and quantified as 1D time-series wavefront slope data. Such high-precision data acquisition capability allows for the simultaneous, real-time capturing of dynamic target characteristics, effectively overcoming the low acquisition efficiency of traditional seismic remote sensing in complex environments.

Consequently, the direct input to our proposed network is this highly sensitive, 1D time-series slope data. Accurately denoising this slope data is of paramount importance, as any residual noise or algorithm-induced phase distortion will be exponentially amplified during the subsequent integration process to reconstruct the true physical amplitude. In recent years, the widespread adoption of Unmanned Aerial Vehicle (UAV) platforms has effectively expanded the spatial detection boundaries of this technology, enabling large-scale, highly maneuverable aerial inspections [4]. However, during actual field flight missions, airborne sensors are exposed to harsh physical environments, and the acquired raw vibration signals are highly susceptible to contamination by complex nonlinear colored noise (e.g., rotor mechanical vibrations, high-frequency wind noise, and atmospheric turbulence). To ensure the acquisition of valid data, high-quality denoising of these massive signals is crucial. Yet, in authentic field exploration scenarios, constrained by portability and battery life, on-site personnel are usually equipped only with portable devices featuring limited computational power (e.g., without independent GPUs). Existing engineering workflows predominantly adopt a “Blind Sampling followed by Offline Processing” mode: UAVs continuously collect massive amounts of raw data, which are then brought back to a high-performance computing laboratory for post-mission cleaning. This long-cycle operational mode carries high engineering risks; if the data is found unusable during post-processing due to excessive environmental noise or sensor baseline drift, it results in substantial losses of time and financial costs.

Furthermore, although recent studies have attempted to transmit raw signals back to cloud servers via wireless networks for online processing (Cloud Computing), real field mapping operations often penetrate deep into remote hinterlands. The system frequently faces “Communication-Denied” dilemmas characterized by a lack of cellular network coverage, severe signal obstruction, or limited uplink bandwidth, making the real-time offloading of massive high-frequency wavefront signals to the cloud difficult to achieve stably. Therefore, reducing the reliance on cloud communication links and remote computing clusters, and exploring lightweight algorithms capable of ultra-fast signal cleaning on computationally restricted portable field devices, as an engineering proof-of-concept toward “In Situ Verification,” has become the key to breaking through the application bottlenecks of this industry.

In response to this demand, researchers initially explored traditional digital signal processing algorithms. However, these methods fundamentally rely on linearity and stationarity assumptions. When dealing with the non-stationary, broadband complex noise generated during dynamic UAV flights, traditional filters are prone to inducing phase distortion and causing the loss of high-frequency harmonics that carry critical physical information. On the other hand, although Empirical Mode Decomposition (EMD) possesses a certain degree of adaptability in nonlinear signal analysis, its recursive Sifting mechanism introduces extremely high computational latency, making it difficult to meet the demands for the rapid on-site processing of tens of thousands of data samples generated in a single flight.

While airborne non-contact wavefront sensing inevitably introduces complex platform noises (e.g., rotor vibrations and aerodynamic turbulence), it is necessary to weigh its costs and benefits against alternative techniques, such as using UAVs to affix clamp-on accelerometers with local storage. From an instrumentation perspective, contact sensors physically bypass fundamental hovering noises, thereby avoiding fundamental signal-noise ambiguities. However, they present significant operational limitations. Firstly, mechanical clamping is operationally risky and often prohibited in hazardous environments involving high-voltage equipment, extreme temperatures, or rotating machinery. Moreover, for structures located in remote or complex terrains, the two-stage logistical process of “deployment and subsequent retrieval” is highly vulnerable to unexpected weather changes, structural occlusions, and communication loss, severely increasing the risk of sensor abandonment and permanent data loss. Secondly, contact sensors only provide discrete, point-wise measurements, lacking the high-resolution, full-field spatial deformation mapping naturally offered by wavefront sensing. Finally, affixing physical modules introduces mass-loading effects, which can significantly alter the intrinsic dynamic properties of lightweight or flexible structures. Therefore, despite the immense challenge of algorithmic denoising, the benefits of non-intrusive, zero-mass-loading, and full-field single-pass inspection make non-contact wavefront sensing an irreplaceable paradigm. The proposed SWIFT-Mamba framework is designed precisely to bridge this gap, mitigating the fundamental noise penalties to unlock the full potential of non-contact edge sensing.

In recent years, the introduction of deep learning technologies has significantly enhanced the high-fidelity denoising performance of wavefront signals. By stacking deep Convolutional Neural Networks (CNNs), Recurrent Neural Networks (RNNs), or Transformer architectures, models can effectively learn complex time-frequency mapping patterns from massive data. In particular, recently proposed physics-inspired dual-channel decoupled networks, such as PRISM [5], have achieved high-quality signal reconstruction under extreme Signal-to-Noise Ratios (SNRs) by jointly extracting amplitude and phase in the complex domain. However, these compute-intensive models universally face challenges of high computational overhead. Complex global attention mechanisms or deep feature pyramids drive the model parameter counts to the millions, keeping Floating Point Operations (FLOPs) persistently high. This renders such high-precision models highly dependent on server-grade computing resources; once deployed on field edge devices, their single-sample inference latency increases significantly. The irreconcilable contradiction between computational constraints and high model precision remains a formidable challenge for the deployment of large networks on edge terminals.

To break the “Pareto Frontier” between computational power and precision, this paper proposes an ultra-lightweight, fast time-frequency state space model tailored for edge computing: SWIFT-Mamba. This paper aims to provide an efficient algorithmic foundation for resource-constrained edge devices, rather than directly demonstrating a mature airborne hardware system. SWIFT-Mamba innovatively introduces the hardware-aware parallel scanning advantages of the One-Dimensional State Space Model (1D-SSM) into wavefront signal processing. While maintaining extremely low parameter counts and computational complexity, the model achieves a favorable trade-off between reconstruction fidelity and edge-oriented fast inference capability through the combined effects of time-frequency dual-track decoupled modeling and a physical-boundary Soft-Clipping mask.

The main contributions of this paper can be summarized in the following three aspects:

1. Physics-Informed Architecture Design: We propose SWIFT-Mamba, a novel end-to-end complex domain enhancement framework. Rather than a trivial integration of existing modules, it introduces a highly synergistic Dual-Track Bottleneck that orthogonally combines deep 1D-SSM (for long-range temporal lag) with asymmetric large-kernel convolutions (for local harmonic topology) at the highly abstracted semantic level.
2. Novel Physical-Boundary Soft-Clipping: To overcome the numerical singularities of traditional complex masking and the severe transient truncation caused by conventional activations (e.g., Tanh or Hard-Clipping), we formulate a novel Soft-Clipping mechanism. It guarantees smooth gradient backpropagation and strict boundary protection for high-fidelity wavefront reconstruction.
3. Phase-Aware Complex Domain Adaptation: We successfully adapt the Mamba sequence modeling paradigm for complex domain optical interferometry. By strictly preserving both magnitude and phase through the Complex Ideal Ratio Mask (cIRM), the framework prevents waveform structural distortion, setting a new benchmark for non-contact wavefront sensing in dynamic airborne environments.

Furthermore, to ensure reproducibility and community accessibility, we have deployed an interactive web demonstration (https://swift-mamba.streamlit.app, accessed on 8 August 2026) that allows external users to independently test the SWIFT-Mamba algorithm on custom or provided sample datasets.

## 2. Related Work

### 2.1. Evolution of Wavefront Sensing Signal Denoising Algorithms

In the field of laser wavefront sensing and precision vibration detection, extracting high-fidelity weak physical signals from strong background noise has consistently remained a core challenge. Early signal denoising methods primarily relied on traditional digital filtering and time-frequency analysis techniques, such as the SG filter, Wiener Filter, and Wavelet Transform. Although these methods possess extremely low computational complexity and fast inference speeds, they are highly prone to causing severe phase distortion and the loss of high-frequency harmonic features due to forced smoothing when processing non-stationary, nonlinear colored noise generated during UAV flights. Furthermore, although EMD exhibits strong adaptability in nonlinear signal decomposition, its recursive Sifting process leads to exponentially increasing computational latency, rendering it incapable of meeting the rapid processing demands of massive data [6,7,8].

With the evolution of deep learning technologies, data-driven neural networks have gradually become the mainstream paradigm for wavefront signal denoising. Traditional Convolutional Neural Networks (CNNs, e.g., DnCNN and Vanilla U-Net) and Recurrent Neural Networks (RNNs) incorporating gating mechanisms (e.g., CRN) have been widely applied to the feature mapping of 2D time-frequency spectrograms. To further enhance denoising fidelity under extreme SNRs, recent cutting-edge research has proposed physics-inspired dual-channel decoupling architectures (such as the PRISM network). By jointly extracting the amplitude and phase of wavefront signals in a Cartesian coordinate system, such methods have successfully broken through the performance bottlenecks of single amplitude-spectrum masks. However, in the pursuit of ultimate denoising precision, these compute-intensive models universally fall into a “computational curse.” Whether utilizing deeply stacked complex-valued convolutions or introducing Bidirectional Long Short-Term Memory networks (Bi-LSTM, as in DCCRN-Lite) for temporal modeling [9,10,11,12,13,14], these approaches cause model parameter counts to skyrocket into the millions, with Floating Point Operations (FLOPs) remaining persistently high. The massive model size and exorbitant inference latency make such high-precision models heavily dependent on server-grade GPUs, obstructing their engineering deployment path on resource-constrained portable edge devices in the field.

### 2.2. Edge Computing and State Space Models

To break the computational barriers of high-precision algorithms in field exploration, lightweight network designs tailored for edge devices have garnered extensive attention in the academic community. Traditional lightweight CNNs (e.g., MobileNet) compress parameter counts through depthwise separable convolutions [15,16]; however, when processing wavefront time-frequency signals, their restricted local receptive fields struggle to capture the Doppler evolutionary continuity along the global time axis. On the other hand, while the Transformer architecture achieves a global receptive field via Self-Attention mechanisms, its computational complexity grows quadratically (*O*(*N^2^*)) with sequence length. This not only severely depletes device memory bandwidth but also creates a severe bottleneck for single-sample inference latency.

Recently, architectures based on structured State Space Models (Mamba) have provided a highly promising solution to these challenges. The Mamba model ingeniously combines the physical state mapping of continuous systems with discretized hardware acceleration in its design. Through a Hardware-aware Parallel Scan algorithm, it successfully reduces the computational complexity of global sequence modeling to an ideal linear scale (*O(N)*) while retaining the infinite memory capacity for long sequences characteristic of RNNs [17,18,19]. Inspired by Mamba’s exceptional computational efficiency, this paper introduces it into the wavefront signal processing domain, which involves 2D coupled characteristics. By innovatively combining global 1D Mamba time scanning with asymmetric wide-kernel frequency convolutions, this paper achieves the efficient orthogonal decoupling of time-frequency features with minimal computational overhead. This provides a foundational algorithm that seamlessly integrates “high fidelity” and “edge-oriented fast inference” performance for in situ verification upon UAV landing.

## 3. Methodology of SWIFT-Mamba

**Figure 1:**
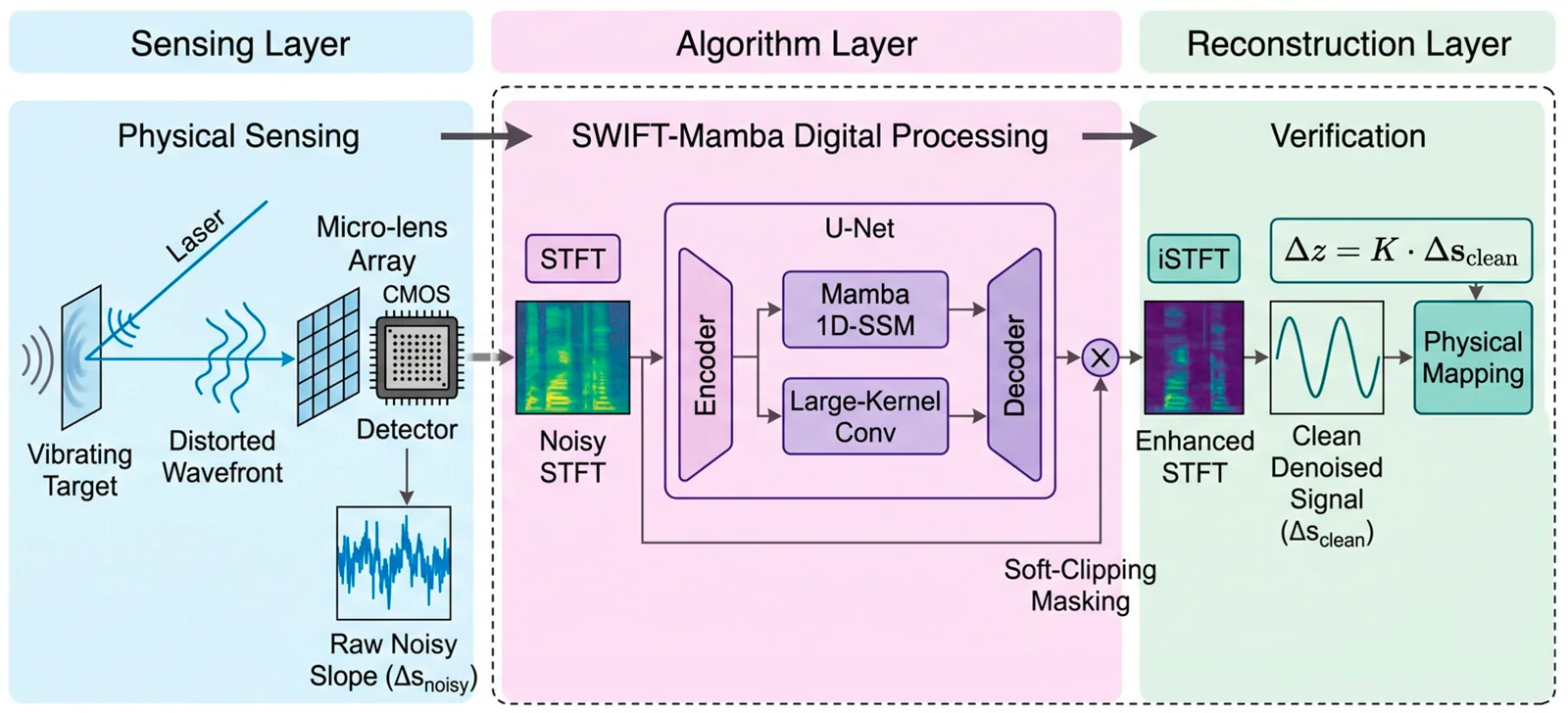
The overall system-level pipeline. The diagram illustrates the complete physical-digital cross-domain workflow, encompassing the initial optical sensing of dynamic wavefront distortions, the digital enhancement via the time-frequency dual-track network, and the final high-fidelity reconstruction of the physical vibration amplitude.

Before detailing the deep learning architecture, it is imperative to establish how the proposed SWIFT-Mamba fits into the overarching physical-digital cross-domain pipeline. As illustrated in Figure 1, the comprehensive workflow is structured into three distinct stages: the Sensing Layer, the Algorithm Layer, and the Reconstruction Layer.

Sensing Layer (Physical Measurement): The physical measurement relies on the geometric optics of the Shack-Hartmann wavefront sensor. When the target surface undergoes a vertical displacement (Δz) due to mechanical vibration, it alters the optical path length of the reflected laser beam. This distorted wavefront passes through a micro-lens array, causing the focal spots to shift by a lateral distance (Δs) on the CMOS detector. Under the small-angle approximation, there exists a strict linear proportionality between the physical surface amplitude and the spot displacement: Δz = *K*·Δs, where *K* is the intrinsic system conversion factor determined by the micro-lens focal length and the receiving telescope’s magnification. By tracking these spot centroids, the system continuously outputs the 1D raw, noise-corrupted slope data (Δs_noisy_).

Algorithm Layer (Digital Processing): To extract the true signal from severe environmental interference, the raw noisy slope (Δs_noisy_) is first transformed via Short-Time Fourier Transform (STFT) into a 2D Noisy STFT spectrogram. This tensor is then fed into the SWIFT-Mamba backbone, featuring a U-Net topology. The encoded features pass through a core Time-Frequency Dual-Track Bottleneck—comprising parallel Mamba (1D-SSM) and Large-Kernel Conv modules—to orthogonally decouple global temporal dependencies and local harmonic structures. The decoder subsequently generates a Soft-Clipping Mask, which is element-wise multiplied (⊕) with the original Noisy STFT to yield the Enhanced STFT.

Reconstruction Layer (Verification): Finally, the Enhanced STFT undergoes an Inverse STFT (iSTFT) to reconstruct the high-fidelity, 1D clean slope sequence (Δs_clean_). Through the final physical mapping (Δz = *K*·Δs_clean_), the system accurately recovers the true physical surface amplitude (Δz), establishing a closed-loop paradigm for edge-oriented in situ verification.

Recently, state-space models (e.g., Mamba) have demonstrated considerable efficacy in sequence modeling owing to their capacity to capture long-range dependencies within linear time complexity. However, naively integrating Mamba blocks as “plug-and-play” components into traditional U-Net architectures (i.e., vanilla U-Net + Mamba) presents notable physical limitations when applied to authentic UAV wavefront signals. On the one hand, black-box mapping within a single time domain struggles to decouple high-frequency mechanical vibrations from the inherent transient variations in the signal. On the other hand, the conventional complex domain multiplication, typically required to process frequency-domain features, substantially increases the computational overhead, which contradicts the lightweight constraints of edge devices.

**Figure 2:**
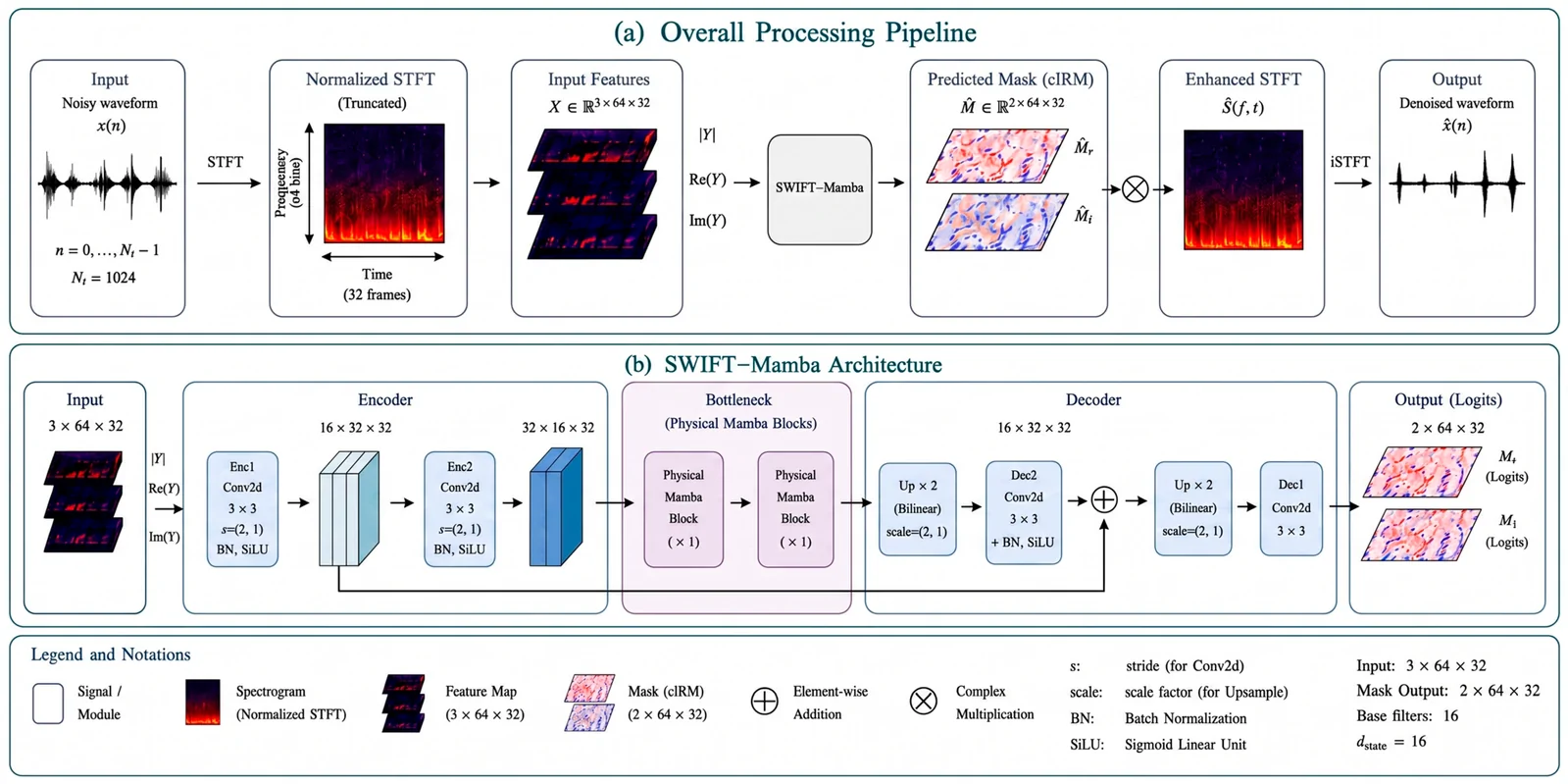
Overall Data Flow and SWIFT-Mamba Network Architecture.

To address these bottlenecks, this study departs from trivial module integration strategies and introduces SWIFT-Mamba, an architecture deeply customized based on the low-level physical manifolds of wavefront signals. A primary distinction from the vanilla U-Net + Mamba framework is the proposed Time-Frequency Dual-Track parallel processing mechanism. This design enables the 1D-SSM to focus exclusively on extracting long-range temporal manifolds, while delegating the processing of frequency-domain harmonic distortions to an independent branch. Furthermore, the proposed model bypasses computationally demanding full complex domain mappings by introducing a Soft-Clipping mask. At a minimal computational cost (with a total parameter count of only 0.066 M), this mechanism achieves precise truncation of physical boundaries while preserving intact phase information. The overall network architecture of SWIFT-Mamba is illustrated in Figure 2.

### 3.1. Overall Signal Processing Pipeline

The overall signal processing and feature mapping pipeline of SWIFT-Mamba is illustrated in Figure 2a. Given the non-stationary nature of wavefront vibration signals under severe noise interference, this model employs the Short-Time Fourier Transform (STFT) to map the 1D time-domain signals into a 2D complex time-frequency domain for enhancement.

Specifically, given a noisy 1D discrete-time waveform *x*(*n*) of length *N_t_* = 1024, where *n* denotes the time sampling index, the system first applies the STFT operation and truncates the core frequency band to construct the input feature tensor *X*. Along the channel dimension, this tensor comprises three physical components: the magnitude spectrum |*Y*|, the complex real part *Re*(*Y*), and the complex imaginary part *Im*(*Y*).

The feature tensor *X* is subsequently fed into the SWIFT-Mamba backbone network, which outputs the predicted cIRM $\overset{ˆ}{M}$. This mask contains both a real-part mask and an imaginary-part mask. Next, the predicted mask is applied to the original noisy complex spectrum via Complex Multiplication to obtain the enhanced clean complex spectrum $\overset{ˆ}{S}\left(f,t\right)$, where *f* and *t* represent the frequency and time frame indices, respectively. Finally, the inverse Short-Time Fourier Transform (iSTFT) is utilized to reconstruct the signal, outputting the denoised 1D time-domain waveform $\overset{ˆ}{s}\left(n\right)$ [20].

### 3.2. Backbone Architecture of SWIFT-Mamba

The backbone feature extraction network of this model adopts a lightweight U-Net topology, as illustrated in Figure 2b. From left to right, the network consists of three main components: an Encoder, a Physical Mamba Bottleneck, and a Decoder.

1. Downsampling Encoder: This component consists of two cascaded convolutional modules (Enc1, Enc2). Each layer comprises a 2D convolution (Conv2d) with a kernel size of 3 × 3, Batch Normalization (BatchNorm), and a SiLU activation function [21,22]. To strictly preserve the high resolution of the frequency axis while compressing the time sequence length, both convolutional layers employ an asymmetric stride operation of *s* = (2, 1). The feature tensor is sequentially downsampled from the initial 3 × 64 × 32 to 16 × 32 × 32, and subsequently to 32 × 16 × 32.
2. Bottleneck: The 32 × 16 × 32 feature map, located at the deepest layer of the network, is fed into the cascaded Physical Mamba Blocks. These blocks accomplish the deep decoupling and extraction of time-frequency features without altering the spatial dimensions of the feature map.
3. Upsampling Decoder: This includes two decoding modules. The feature map first recovers its spatial resolution via Bilinear Upsampling combined with a scale factor of *scale* = (2, 1), followed by a 3 × 3 convolution for feature reconstruction. To alleviate the loss of high-frequency details caused by downsampling, the model introduces a skip connection via element-wise addition (⊕) between the first-layer decoding output (Dec2) and the first-layer encoding feature (Enc1). Furthermore, a learnable adaptive weight coefficient α is introduced:
  (1) $$F_{fuse}=F_{dec2}+\alpha \cdot F_{enc1}$$
  where *F_dec_*_2_ denotes the upsampled features from the decoder, *F_enc_*_1_ represents the shallow features from the encoder, and α is a scalar weight automatically optimized by the network during the training process. This weight serves to dynamically balance shallow details with deep semantics. Ultimately, the network outputs an unbounded Logits tensor with dimensions of 2 × 64 × 32.

### 3.3. Time-Frequency Dual-Track Physical Mamba Module

**Figure 3:**
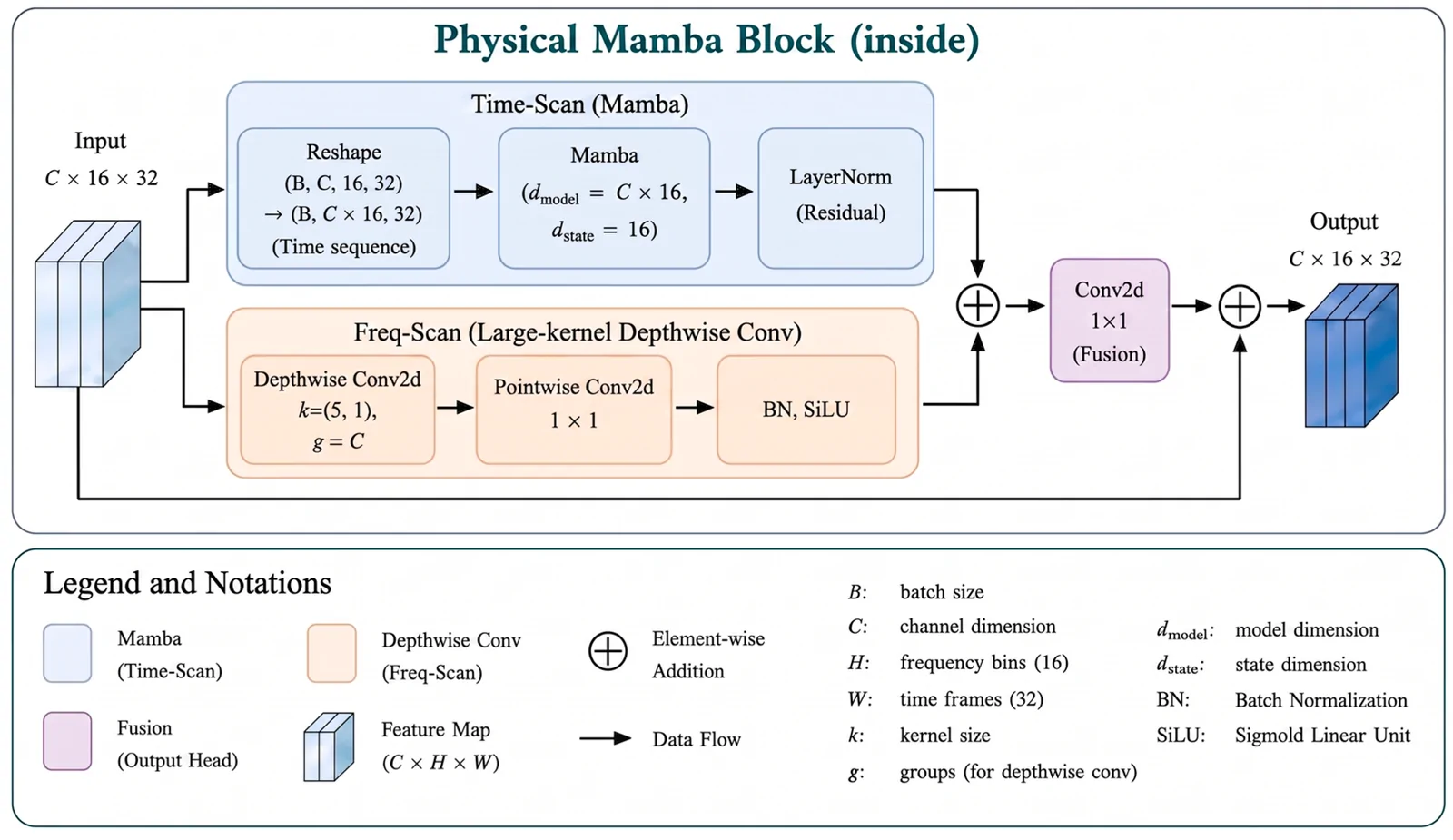
Internal Structure of the Physical Mamba Module.

If traditional Mamba models directly process flattened 2D time-frequency spectrograms, they inevitably destroy the harmonic topology of the frequency axis. To address this, this paper designs the Physical Mamba Block, whose internal structure is illustrated in Figure 3. Through a parallel time-frequency dual-track mechanism, this module achieves multi-dimensional physical feature capture with linear *O*(*N*) complexity.

#### 3.3.1. Time-Scan Path (Time-Scan Mamba)

This branch is designed to capture the Doppler phase shifts and long-range dependencies of the signal as it evolves over time. The input feature map of size C × 16 × 32 is first transformed into a sequence format (B, C × 16, 32) via a Reshape operation. Within the core Mamba State Space Model, the continuous-time physical dynamic system is discretized using a step size parameter Δ into the following iterative process:

(2) $$\bar{A}=\exp\left(\Delta A\right)$$

(3) $$\bar{B}={\left(\Delta A\right)}^{-1}\left(\exp\left(\Delta A\right)-I\right)\cdot \Delta B$$

(4) $$h_{t}=\bar{A}h_{t-1}+\bar{B}x_{t}$$

(5) $$y_{t}=Ch_{t}$$

where Δ denotes the discretization step size parameter utilized to map the continuous system into a discrete system; *A*, *B*, and *C* represent the system state and projection matrices of the continuous-time state space model; $\bar{A}$ and $\bar{B}\;$are the discretized state transition matrix and input matrix, respectively; *x_t_* is the input sequence feature vector at time step *t*; *h_t_* and *h_t−_*_1_ denote the hidden state vectors at the current and previous time steps; and *y_t_* is the output response feature generated at the current time step.

#### 3.3.2. Minimalist Frequency-Aware Path (Freq-Scan Path)

To prevent large-kernel convolutions from contaminating the temporal receptive field, this branch introduces a depthwise separable convolution. Its kernel size is asymmetrically set to *k* = (5, 1), with the number of groups defined as *g = C* (i.e., depthwise convolution). This operation exclusively performs local harmonic scanning across 5 frequency bins along the frequency axis, with the temporal span strictly constrained to 1. Subsequently, a 1 × 1 point-wise convolution is employed to facilitate cross-channel information interaction.

Ultimately, the time-frequency dual-track features are fused via channel concatenation, reduced in dimensionality through a 1 × 1 convolution, and combined with a Global Residual to yield the final module output.

### 3.4. Physical-Boundary Soft-Clipping and Complex Mask Construction

**Figure 4:**
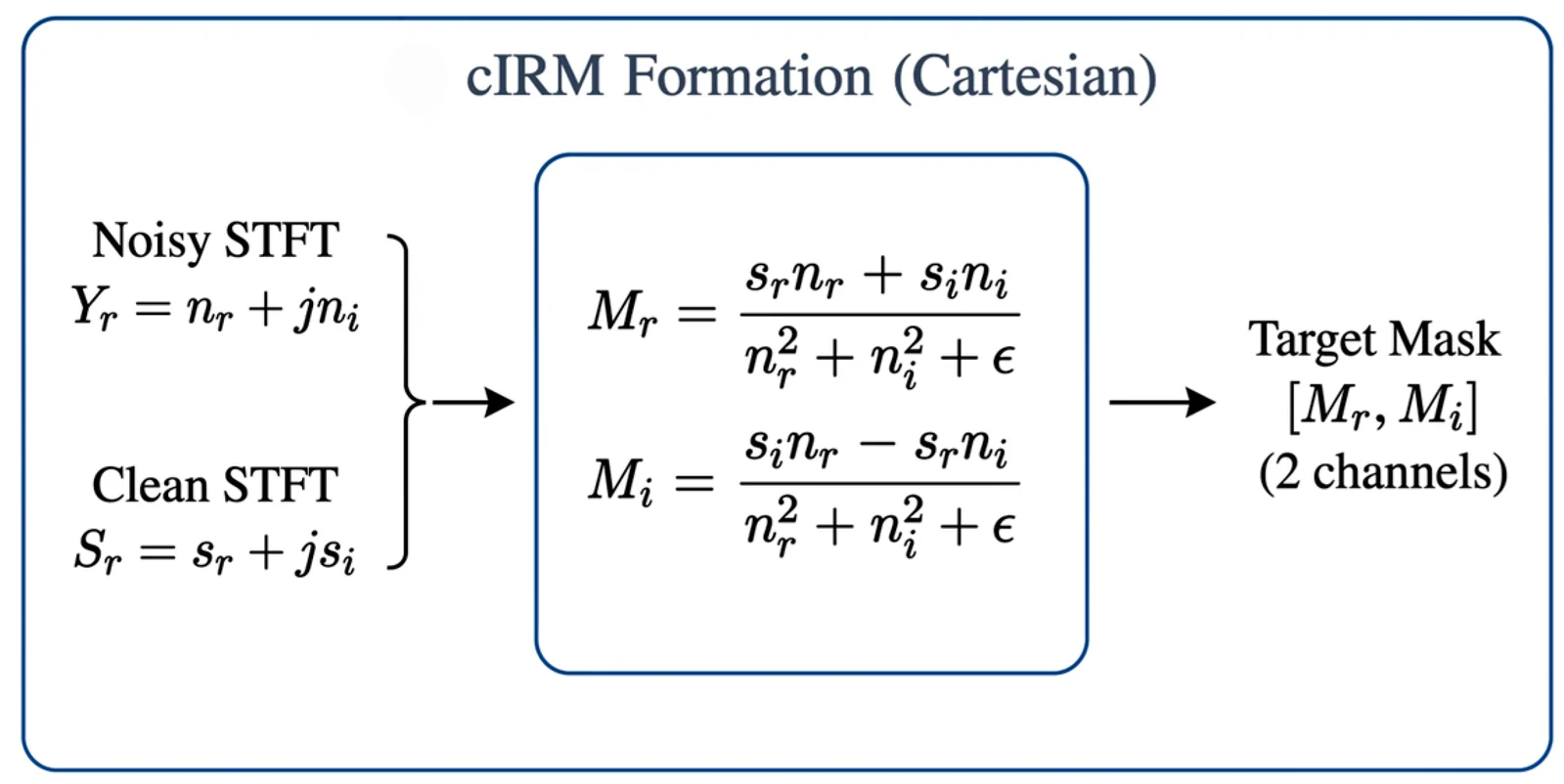
cIRM Architecture.

In the complex domain, the core objective of the system is to reconstruct the cIRM. As illustrated in Figure 4, let the clean STFT signal be denoted as *S_r_ = s_r_ + j s_i_*, and the noisy STFT signal as *Y_r_ = n_r_ + j n_i_*. In the Cartesian coordinate system, the theoretical real part *M_r_* and imaginary part *M_i_* of the cIRM are calculated as follows:

(6) $$M_{r}=\frac{s_{r}n_{r}+s_{i}n_{i}}{n_{r}^{2}+n_{i}^{2}+\epsilon }$$

(7) $$M_{i}=\frac{s_{i}n_{r}-s_{r}n_{i}}{n_{r}^{2}+n_{i}^{2}+\epsilon }$$

where *s_r_* and *s_i_* represent the real and imaginary parts of the clean target signal’s STFT, respectively; *n_r_* and *n_i_* denote the real and imaginary parts of the noisy input signal’s STFT, respectively; and ϵ is a small positive real constant (e.g., 10^−8^) introduced to prevent computational overflow caused by a zero denominator [23].

**Figure 5:**
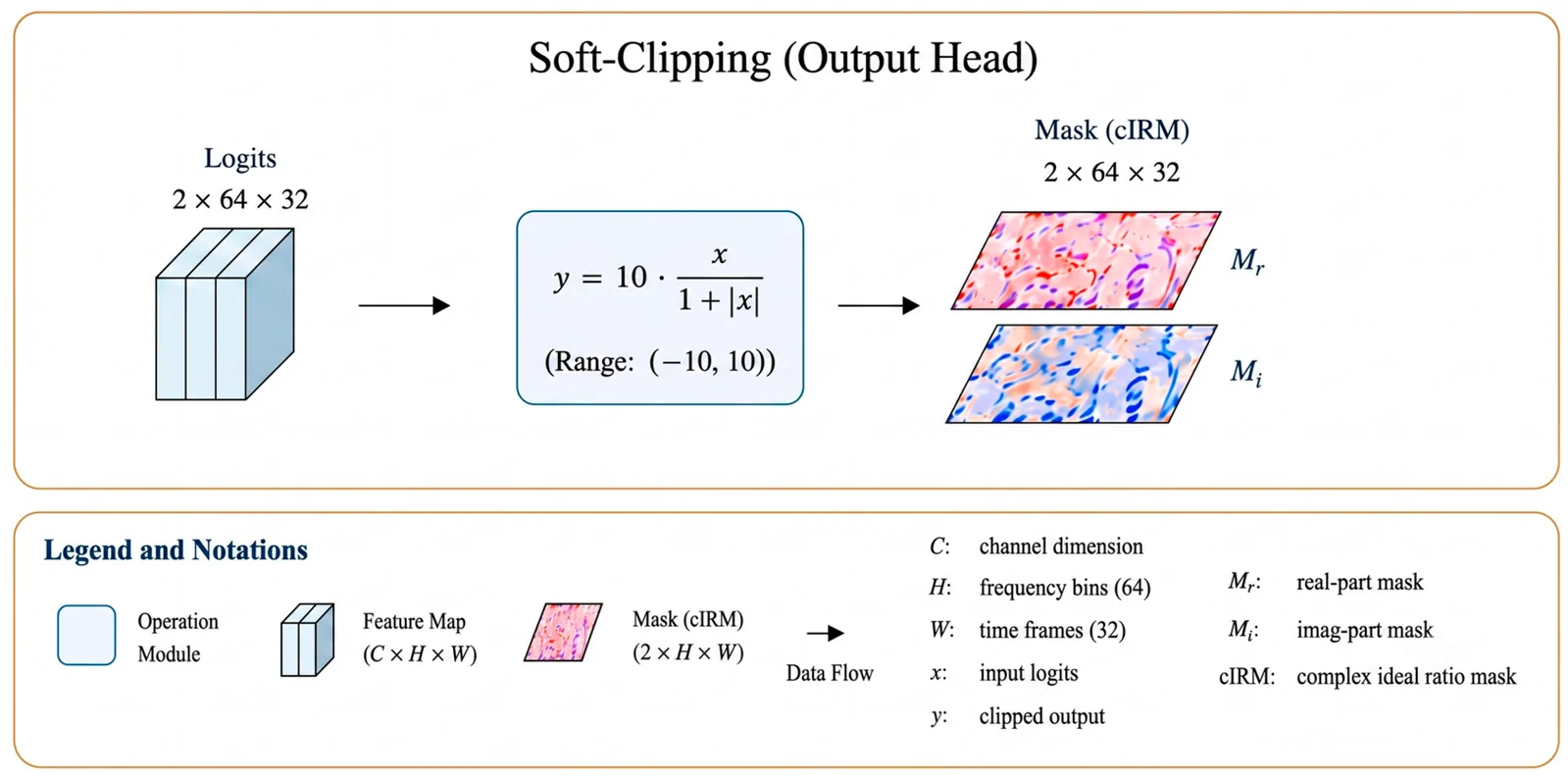
Soft-Clipping Mechanism for Physical Boundaries.

Under extreme operating conditions, the noise energy in specific frequency bands may approach zero, causing the target mask values to tend towards infinity; direct prediction would thus trigger severe gradient explosion. To address this, this paper designs a physical-boundary Soft-Clipping mechanism at the output end, as illustrated in Figure 5. This clipping mechanism is continuously differentiable, which not only ensures the gradient stability of the full complex domain mapping but also preserves the backpropagation efficiency of the deep neural network, without disrupting the original scientific computing functional modules. The raw dual-channel unbounded Logits tensor *x* output by the decoder must be mapped into the mask value *y* via the following nonlinear activation function:

(8) $$y=10\cdot \frac{x}{1+\left|x\right|}$$

where *x* represents any real-valued prediction output by the network; *y* is the clipped mask amplitude applied to the complex multiplication; and the constant 10 denotes the predefined absolute physical boundary upper limit. This function ensures that the predicted values maintain a linear gradient near zero, while infinitely smoothly and strictly asymptoting to the (−10, 10) boundary as the absolute value increases, thereby eradicating the parasitic spike phenomenon at its root.

### 3.5. Differences from Existing Architectures

Although the proposed SWIFT-Mamba macroscopically draws inspiration from the multi-scale concept of U-Net and the linear scanning mechanism of the State Space Model (SSM), its core design is not a trivial plug-and-play assembly of existing modules. Instead, it is a specific reconstruction deeply tailored to the physical evolution characteristics of UAV-borne wavefront slope signals. The essential differences are manifested in the following three dimensions:

1. Time-Frequency Dual-Track Decoupling vs. Traditional Single-Domain Modeling: The standard Mamba architecture was originally designed for Natural Language Processing (NLP), treating inputs as discrete single-domain 1D sequences. However, physical mechanical vibrations not only encompass transient evolution and temporal lag in the time domain but also contain robust harmonic topological structures in the frequency domain. Directly embedding Mamba blocks into a conventional U-Net would lead to a severe loss of frequency-domain topological information. The time-frequency dual-track sensing paths (Time-Scan and Freq-Scan) designed in this paper force the network to explicitly decouple the physical manifolds of these two dimensions during the feature extraction stage, thereby achieving precise stripping of non-stationary colored noise with an extremely low parameter count.
2. Physical Synergy of Asymmetric Convolutions and SSM vs. Pure State Space Scanning: Unlike natural language tokens, which lack continuous physical spatial correlation, wavefront slope signals exhibit high physical continuity within local time windows. Therefore, instead of employing pure Mamba blocks, this study prepends asymmetric large-kernel convolutions (e.g., *k* = (5, 1)) before the global SSM scanning. This design utilizes convolutions to rapidly establish a local physical receptive field, followed by the SSM to capture global long-range dependencies. This effectively prevents the local high-frequency glitch phenomenon that pure Mamba architectures are prone to when processing continuous waveform signals.
3. Physical-Boundary Soft-Clipping Mask vs. Conventional Activation Functions: Standard deep learning denoising networks typically output in the real number domain and rely on conventional activation functions such as Tanh or Sigmoid. Addressing the critical pain point that wavefront signals are highly susceptible to phase fracturing in the complex domain, SWIFT-Mamba does not directly output the reconstructed sequence. Instead, it predicts a cIRM innovatively coupled with a physical-boundary Soft-Clipping mechanism. This replaces conventional hard-clipping from the underlying physical logic, eliminating numerical singularities caused by complex division and providing robust topological protection for high-frequency transient peaks under extreme conditions.

## 4. Experimental Setup and Evaluation Metrics

To validate the efficacy and lightweight advantages of the proposed SWIFT-Mamba in wavefront vibration signal enhancement tasks, this section details the construction process of the experimental dataset, the hyperparameter settings for core network training, and the evaluation metrics. Furthermore, it comprehensively analyzes the model’s denoising performance under various operating conditions.

### 4.1. Dataset Construction and Preprocessing

**Figure 6:**
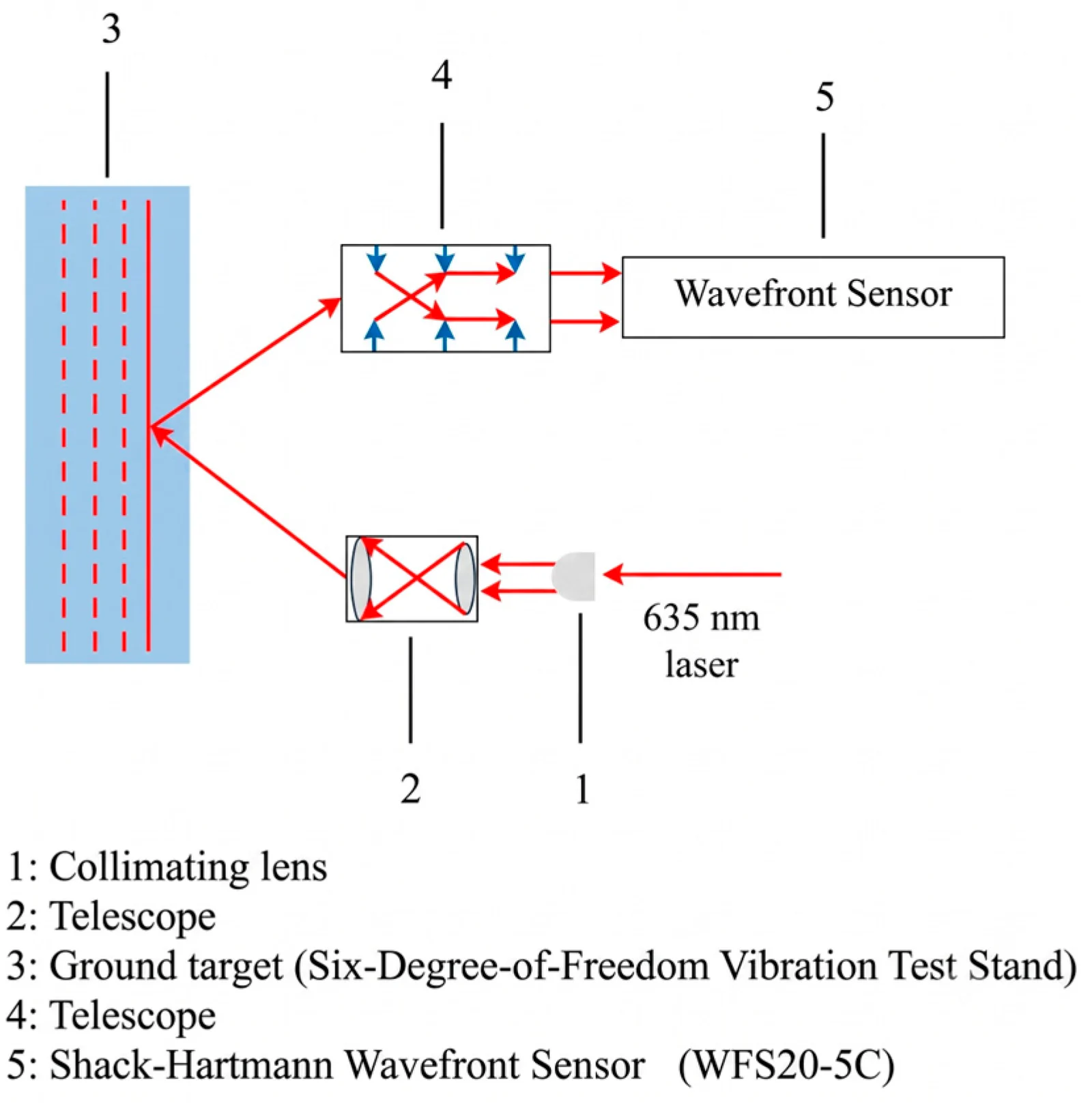
Schematic diagram of the complete vibration signal acquisition experimental system. The solid and dotted red lines denote the static reference position and the dynamic displacement boundaries of the vibrating target, respectively. Red arrows trace the laser’s optical path, while blue arrows represent convex lenses.

This study utilizes a massive dataset of wavefront slope vibration signals acquired via a laser remote sensing seismic wave detection system. The complete experimental system setup is illustrated in Figure 6. In terms of data acquisition hardware and software configuration, the system employs a 70 mW continuous-wave laser to conduct high-sensitivity detection at a distance of approximately 10 m. The core detection component is a Thorlabs Shack-Hartmann wavefront sensor (Model: WFS20-5C, Thorlabs GmbH, Dachau, Germany), which features a wavefront reconstruction accuracy of up to λ/30 (RMS@633 nm) and is coupled with a CMOS image sensor (software version: 18183-D03). The experiment conducts real-time monitoring via the LabVIEW software platform (LabVIEW 2020.0 (32-bit)) and adopts a high-speed sampling mode of 800 fps to obtain the wavefront slopes at the centroids of the sensor’s 6 × 6 micro-lens array. Simultaneously, the system utilizes a six-degree-of-freedom controlled vibration test stand to precisely regulate the amplitude and frequency of the target vibration.

Regarding the pairing of clean and noisy samples, it is physically impossible to simultaneously capture perfectly synchronized, strictly paired ‘clean’ and ‘noisy’ signals of the exact same vibration event in an open field due to the inherent stochasticity of atmospheric turbulence and ambient light. Therefore, we adopted a semi-synthetic paired training strategy. The ‘clean’ ground-truth signals were acquired exclusively at night within a completely sealed shielding enclosure, strictly isolating airflow and stray light. The ‘pure noise’ profiles were recorded during the daytime without the enclosure, capturing authentic environmental background clutter. The paired training samples were then mathematically synthesized by mixing these independent high-fidelity clean signals with the real-world pure noise profiles. A total of over 60,000 data samples were collected under various discrete frequencies (0.1, 0.15, 0.18, and 0.2 Hz) and multi-gradient vibration amplitudes (0.50, 0.62, 0.75, 0.81, 0.87, 0.93, 1.00, 1.12, and 1.18 mm).

The length of each individual data sample is strictly fixed at 1024 sampling points. The dataset was partitioned into training, validation, and testing sets with an 8:1:1 ratio. Crucially, to prevent data leakage and avoid over-optimistic evaluations, we enforced a strict temporal-isolation splitting protocol. Signal segments extracted from the same continuous acquisition session (i.e., a single continuous vibration event generated by the test stand) were strictly confined to a single split. No samples from the same acquisition timeline were allowed to appear simultaneously in the training and testing sets. This ensures that the test set evaluates the model’s true algorithmic generalization to unseen physical vibration events, rather than merely memorizing localized environmental noise states. Specifically, the training set comprises 52,338 randomly selected clean signal samples alongside 8067 pure noise samples. To comprehensively and rigorously evaluate the model’s generalization capability and robustness under actual, complex, and uncontrolled operating conditions, the test set is further divided into two subsets: laboratory-measured signals and synthetic signals. The empirical test set directly utilizes 8152 real noisy signals acquired in the laboratory environment, whereas the synthetic test set is constructed using an independent pool of 3738 clean signals and 1886 pure noise samples. However, we acknowledge that while this additive synthesis provides a strictly controlled mathematical benchmark for evaluating algorithmic components, it does not fully emulate the highly complex, nonlinear aero-optical coupling noises induced by periodic UAV rotor vibrations and severe atmospheric turbulence in authentic airborne environments.

To address the evaluation of these real-world acquisitions, it is essential to strictly define the evaluation baseline for the empirical laboratory dataset. Unlike synthetic data, true airborne-equivalent field acquisitions inherently lack a perfectly phase-aligned, noise-free mathematical ground truth due to unavoidable optomechanical thermal drift and nonlinear mechanical hysteresis between acquisition sessions.

To facilitate a quantitative engineering evaluation, we establish a Hardware-in-the-Loop Pseudo-Ground-Truth (Pseudo-GT). Specifically, pristine signals acquired during strictly shielded nighttime operations (driven by identical programmable vibration commands) serve as the macroscopic physical baseline for the unshielded daytime noisy measurements. While microscopic sub-wavelength phase misalignments inevitably exist—meaning the absolute metric values (e.g., SNR, PCC) on the empirical set should be interpreted as approximate engineering lower bounds rather than strict mathematical limits—this Pseudo-GT methodology provides a highly reliable, physically anchored reference to evaluate the model’s forward denoising robustness in authentic environments.

**Figure 7:**
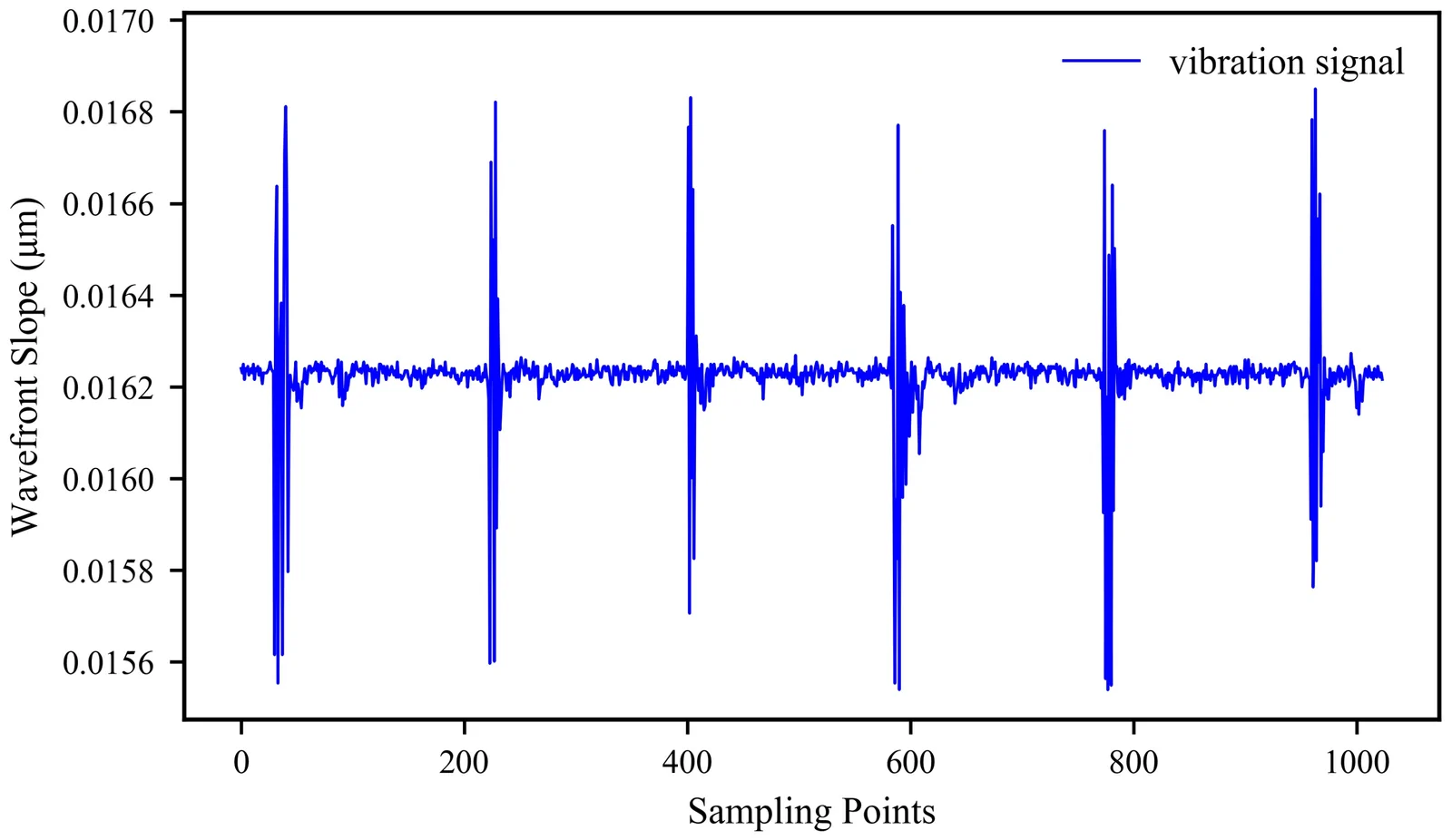
Clean vibration signals acquired in a laboratory setting. Even clean vibration signals acquired behind a physical shield and at night contain some background noise.

**Figure 8:**
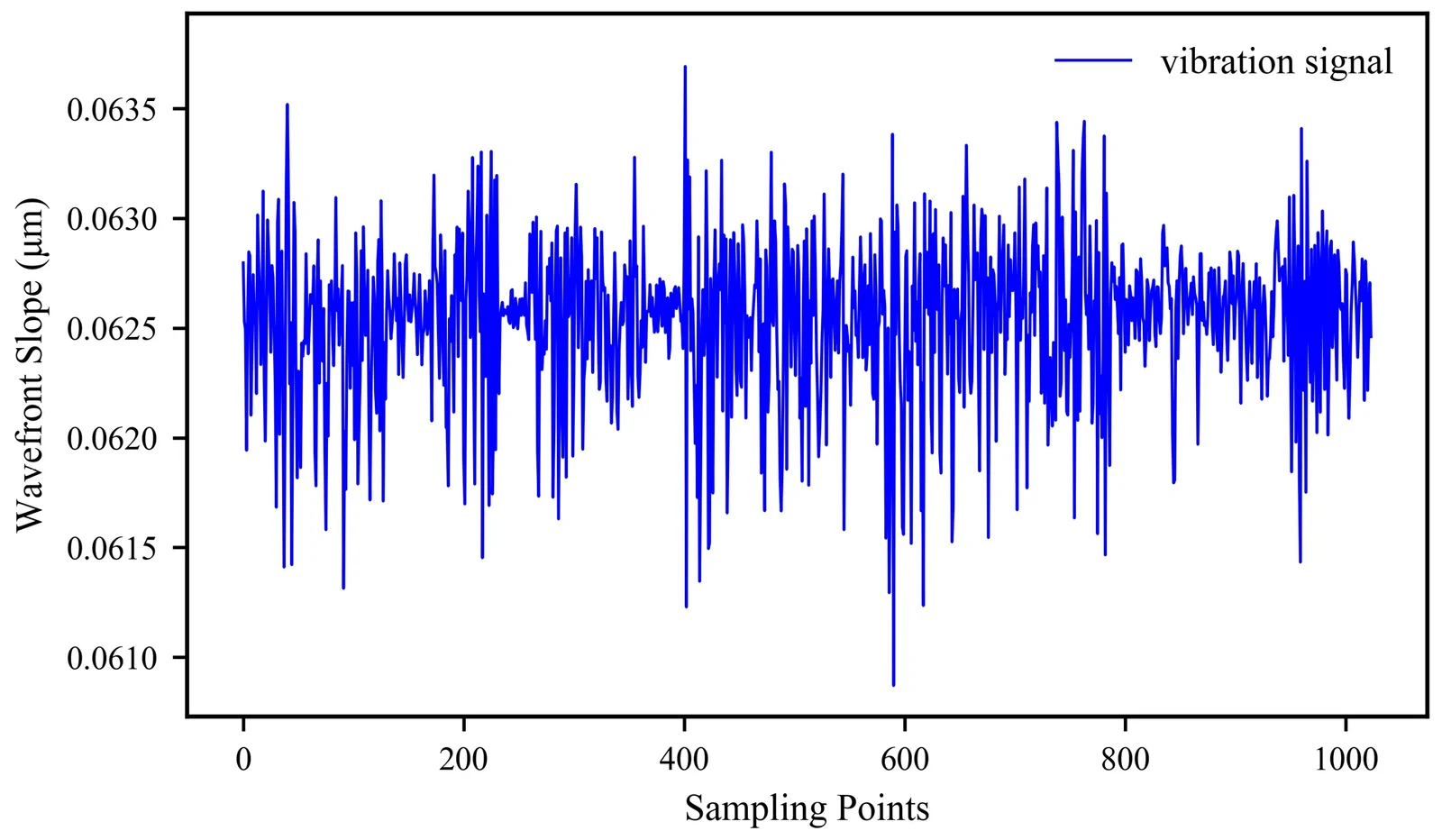
Vibration signal recorded without a physical shield; the vibration signal is drowned out by noise.

The clean signals acquired in the laboratory environment are illustrated in Figure 7, while the noisy signals are depicted in Figure 8.

In both the construction of the synthetic test set and the dynamic data augmentation during the training phase, this paper adopts a controlled noise injection paradigm. Given a clean wavefront vibration signal *s* and a background noise signal *n*, the synthesis formula for the noisy signal *x* is defined as follows:

(9) x=s+K⋅n

where *K* represents the energy scaling coefficient, utilized to precisely control the target SNR of the synthetic signal. During test set generation, three noise samples are randomly selected from a pool of 1886 pure noise samples and superimposed onto each independent clean signal. This process consistently generates 11,214 synthetic noisy cases covering a broad spectrum of SNR levels. During the network training phase, to endow the model with the resilience required to handle highly variable and extreme operating conditions, the target SNR is configured to follow a uniform distribution ranging from [−20, 15] dB. Simultaneously, to prevent the model from overfitting to the mere act of “noise stripping”—which could inadvertently damage the high-frequency manifold of the original signal—a zero-noise injection probability of 15% is integrated into the data loading mechanism. During training, this mechanism randomly feeds clean signals directly as noisy inputs, compelling the network to learn and preserve exceptional identity mapping capabilities.

### 4.2. Experimental Environment and Training Strategy

The experiments in this study are implemented based on the PyTorch deep learning framework [24]. To accommodate both large-scale parallel training and inference robustness across hardware platforms, the network training, deployment, and comparative inference experiments are conducted on an NVIDIA RTX 3090 GPU.

Regarding the training strategy, the AdamW optimizer is employed for parameter updates, incorporating a weight decay mechanism (with a coefficient set to 1 × 10^−4^) to prevent overfitting. To accelerate model convergence within the complex loss space, the OneCycleLR strategy is adopted for learning rate scheduling, with the peak learning rate set to 0.003 and the warm-up phase accounting for 10% of the total steps [25,26,27]. The total number of training epochs is set to 200, with a batch size of 256. An Early Stopping mechanism based on the combined validation loss is integrated during training, featuring a patience of 30 epochs.

### 4.3. Evaluation Metrics

To comprehensively and objectively evaluate the overall performance of the SWIFT-Mamba model, this paper strictly divides the evaluation framework into two dimensions: signal reconstruction quality and lightweight computational efficiency.

#### 4.3.1. Signal Reconstruction Quality Metrics

At the signal recovery level, six widely recognized acoustic and physical metrics are selected [28]. Metrics where higher values indicate better performance are denoted by (↑), whereas those where lower values imply smaller errors are denoted by (↓):

1. Signal-to-Noise Ratio (SNR, ↑): Evaluates the core capability of the network to eliminate background noise, defined as the difference between the SNR of the output denoised signal and that of the noisy input signal.
2. Signal-to-Distortion Ratio (SDR, ↑): Measures the overall physical fidelity in time-domain energy between the denoised waveform and the clean reference waveform [29].
3. Scale-Invariant Signal-to-Distortion Ratio (SI-SDR, ↑): By employing orthogonal projection, it masks the impact of overall signal amplitude scaling, providing a more objective assessment of the reconstruction quality of the waveform’s internal structure and phase. It is currently one of the most fundamental evaluation criteria in the signal separation domain.
4. Pearson Correlation Coefficient (PCC, ↑): Calculates the linear correlation between the denoised signal and the reference signal, reflecting the degree of recovery of the waveform envelope.
5. Normalized Mean Square Error (NMSE, ↓): Precisely quantifies the sample-level absolute energy error between the predicted waveform and the ground-truth waveform.
6. Log-Spectral Distance (LSD, ↓): Computes the Euclidean distance between the log-spectrograms of the denoised signal and the clean signal, reflecting the smoothness of the frequency response and the recovery quality of high-frequency harmonics.

#### 4.3.2. Lightweight and Computational Efficiency Metrics

Considering that wavefront vibration monitoring systems are typically deployed on edge devices with constrained computational resources and memory, this study pursues not only high denoising fidelity but also prioritizes the model’s lightweight deployment potential as a core evaluation dimension. Therefore, this paper discards the model physical size metric, which is easily perturbed by the encapsulation of underlying deep learning frameworks, as well as the throughput metric, which possesses mathematical reciprocal redundancy with latency [30,31]. Instead, it streamlines and establishes the following two complementary core evaluation criteria (both where lower values indicate better performance, denoted by ↓):

1. Parameters (Params, ↓): Quantifies the total number of learnable weights within the model, measured in millions (M). This metric directly determines the theoretical lower bound of memory resources occupied on edge devices during actual deployment, objectively reflecting the network’s level of compactness in terms of spatial complexity.
2. Floating Point Operations (FLOPs, ↓): Measures the total computational volume required for the model to complete a single full forward inference pass, quantified in gigas (G). As a hardware-agnostic, purely physical evaluation metric, FLOPs rigorously quantifies the model’s absolute time complexity and foundational computational consumption at the algorithmic architecture level.

## 5. Experimental Results and Analysis

### 5.1. Intrinsic Performance Evaluation of SWIFT-Mamba

Before benchmarking the proposed model against mainstream deep learning baseline networks, this section conducts a multi-dimensional independent validation of SWIFT-Mamba’s intrinsic signal enhancement capabilities and physical generalization. To comprehensively dissect the model’s scientific reconstruction quality for wavefront vibration signals, we systematically constructed an overall baseline performance test, a varying-amplitude response test at an identical frequency, a varying-frequency response test at an identical amplitude, and a step-wise robustness test under harsh conditions across a wide SNR range.

#### 5.1.1. Overall Denoising Baseline Performance Evaluation

To establish the baseline performance of the SWIFT-Mamba model in the wavefront vibration signal reconstruction task, this section conducts a macroscopic statistical evaluation of its overall average performance across two types of massive datasets. As demonstrated by the metric summary in Table 1, the model exhibits exceptionally stable and superior waveform restoration alongside high-fidelity characteristics under heterogeneous operating conditions.

First, on the real-world laboratory-measured dataset—which incorporates non-stationary mechanical vibrations and environmental turbulence—SWIFT-Mamba exhibited remarkable reconstruction fidelity. Its SI-SDR and SDR gain reached to 16.10 dB and 16.21 dB, respectively, effectively preserving the physical phase and core topology. Concurrently, the NMSE was drastically compressed to 0.0292, the PCC peaked at 98.78%, and the LSD decreased to a mere 0.076 dB. The fact that the model demonstrates such good robustness under real-world, harsh operating conditions proves that it has bridged the “reality gap” and avoided the pitfall of overfitting to controlled noise that often results from a test-driven approach. Subsequently, on the controlled synthetic signals—spanning a broad frequency band and multi-gradient SNRs—the model delivered an outstanding SNR gain of 19.82 dB, demonstrating a robust background noise stripping capability. Its PCC (98.55%) and LSD (0.080 dB) remained highly congruent with its empirical performance, highlighting the network’s precise anchoring and control over the core harmonic energy distribution, even under violently fluctuating noise coverage.

Synthesizing its exceptional performance across both datasets, this powerful generalization potential—which essentially transcends noise domain discrepancies—can be fundamentally attributed to the model’s time-frequency dual-track decoupling architecture and the physical-boundary Soft-Clipping mechanism. This indicates that SWIFT-Mamba has genuinely captured the underlying physical mechanisms of the wavefront signal’s base manifold, laying a performance foundation for subsequent analyses under extreme operating conditions and edge deployment.

#### 5.1.2. Validation of Scale-Invariance Under Identical Frequency and Varying Amplitudes

In real-world industrial environments and optical interferometry monitoring, the target vibration signals captured by sensors frequently exhibit drastic amplitude fluctuations spanning target vibration amplitudes may vary over a broad dynamic range. These variations arise from differences in the distance to mechanical excitation sources, medium damping attenuation, and optical path oscillations. An industrial-grade enhancement network of practical utility must inherently possess exceptional Scale-Invariance; specifically, it must maintain consistent reconstruction gains for both faint, low-amplitude signals and transient, large-amplitude signals without inducing nonlinear distortion.

To verify this, this experiment fixed the fundamental frequency characteristics of the noisy signal and incrementally increased the dynamic input amplitude from 0.5 mm to 1.18 mm, thereby systematically evaluating the model’s ability to respond to microscopic variations. See Table 2 for specific, item-by-item evaluation metrics.

As shown in Table 2, with the cross-magnitude amplification of the target signal’s physical amplitude, SWIFT-Mamba exhibits exceptional robustness and gain-invariance characteristics across multiple core reconstruction metrics. In the weak amplitude scenario of 0.50 mm, the model’s output signal-to-noise ratio (SNR) consistently maintains a high level of 17.47 dB, and the Pearson correlation coefficient (PCC) reaches an impressive 98.25%. This highly challenging test result robustly verifies that the network possesses superior sensitivity in extracting weak signal features, enabling it to accurately capture effective components under low-SNR boundaries. Consequently, it successfully circumvents the phenomena of “in situ erasure” or “over-smoothing” commonly found in traditional denoising algorithms when the amplitude approaches the noise floor.

When the input amplitude is extended to a high-energy excitation state of 1.12 mm, despite the slight spectral boundary truncation effect induced by transient drastic fluctuations during the short-time Fourier transform (STFT) time-frequency mapping process—which causes the log-spectral distance (LSD) to marginally increase to 0.087 dB—the model’s scale-invariant signal-to-distortion ratio (SI-SDR) remains firmly locked at an ultra-high fidelity level of 16.69 dB. This indicates that the model can completely anchor the phase topology and waveform profile of the signal even when subjected to large-dynamic-range energy impacts.

Such highly consistent performance across complex amplitude fluctuations is fundamentally rooted in the sophisticated architectural mechanism within SWIFT-Mamba. By introducing the physical-boundary Soft-Clipping complex mask at the output stage, the network constrains mask estimation within a physically meaningful range while preserving phase-related information. This mechanism largely eliminates the interference of absolute gain on the nonlinear representation weights of the State Space Model (SSM), forcing the network to focus strictly on the temporal evolution patterns themselves. This empirical physical attribute aligns with the stringent robustness requirements for multi-level, complex amplitude fluctuations in industrial edge-oriented fast inference monitoring tasks.

#### 5.1.3. Validation of Cross-Band Generalization Under Varying Frequencies and Identical Amplitude

Beyond amplitude fluctuations, broadband mechanical failures or multi-stratum acoustic reflections can cause the harmonic energy of wavefront vibration signals to scatter across an extremely broad frequency band. Traditional denoising models based on convolutional local receptive fields, after undergoing successive downsampling and pooling operations, frequently suffer from severe high-frequency phase “flattening” and spectral leakage. To validate SWIFT-Mamba’s capability for the lossless reconstruction of diverse frequency components, this section conducts a comparative evaluation across discrete physical frequency points ranging from the low-frequency to mid-high-frequency bands (0.1 Hz to 0.2 Hz) under a fixed input amplitude. The quantitative test results are presented in Table 3.

The results in Table 3 indicate that SWIFT-Mamba maintains stable reconstruction performance across the tested frequency range. At 0.10 Hz, the model achieves SDR and SI-SDR gain of 16.82 dB and 16.76 dB, respectively. At 0.20 Hz, the SNR gain remains 20.45 dB and the PCC remains 98.90%. These results suggest that the frequency-aware path contributes to preserving frequency-domain structures and that the 1D Mamba scanning path helps model long-range temporal dependencies. In a high-density harmonic region with a frequency of 0.20 Hz, the rate of the signal’s temporal evolution doubles. While traditional Long Short-Term Memory (LSTM) networks are highly susceptible to phase lag or high-frequency attenuation under such conditions, the proposed model robustly sustains an SNR gain of 20.45 dB and an extraordinarily strong PCC of 98.90%. This conclusively validates that the parallel frequency-aware path—featuring an asymmetric large-kernel depthwise separable convolution of *k* = (5, 1) serves as a critical line of defense. Functioning akin to a precise frequency-domain “comb filter,” it accurately anchors the high-frequency Doppler topology across the frequency axis without compromising the resolution of the temporal receptive field, thereby ensuring the lossless reconstruction of all full-band components.

#### 5.1.4. Evaluation of Extreme Anti-Noise Robustness Across a Wide SNR Range

**Figure 9:**
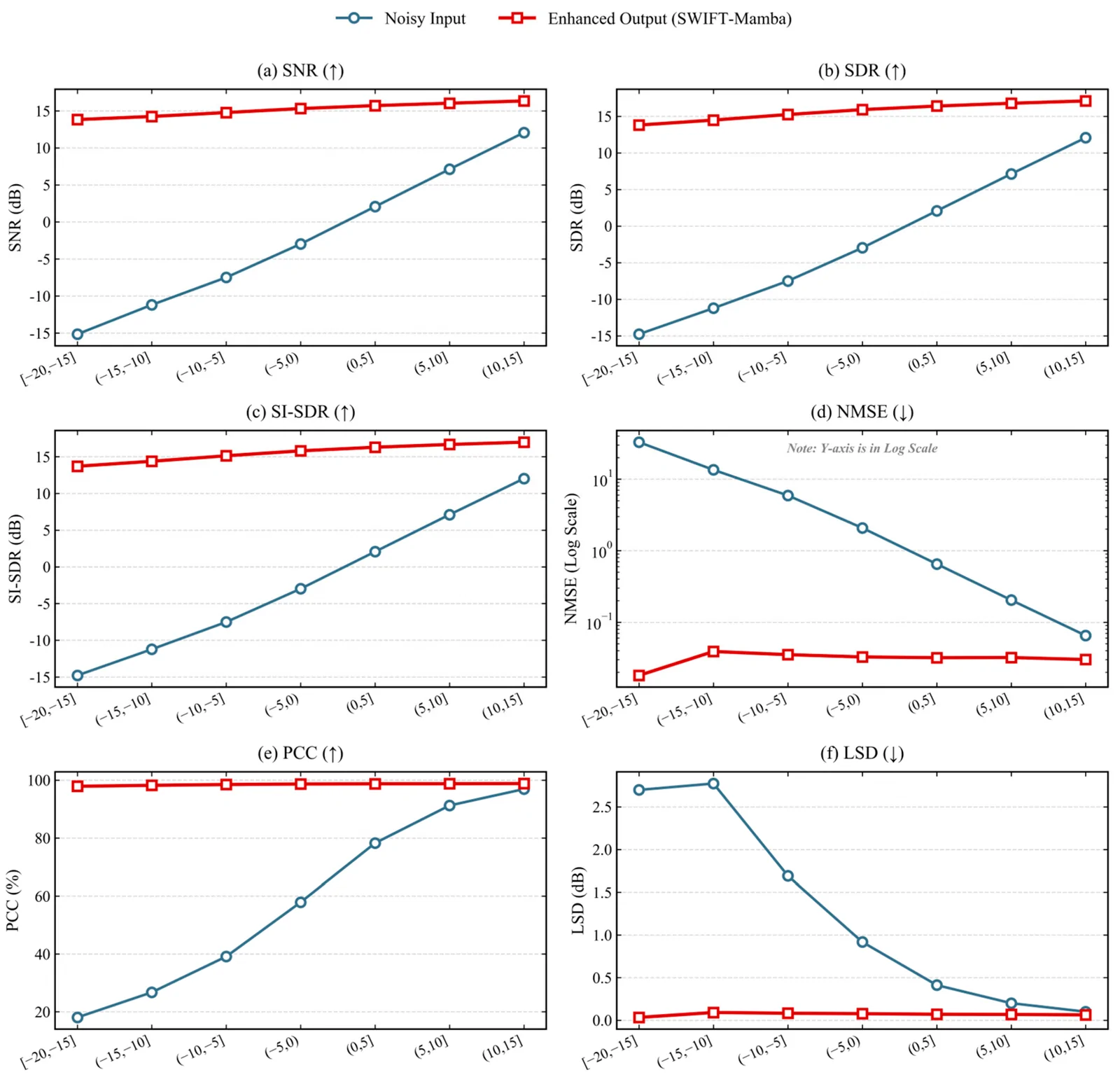
Evaluation metrics for synthetic noisy signals across different SNR intervals. In the captions of the six subplots, ↑ indicates that a higher value is better, while ↓ indicates that a lower value is better. The SNR, SDR, and SI-SDR values shown in the figure are all gain values. The NMSE, PCC, and LSD values are all output values.

**Figure A1:**
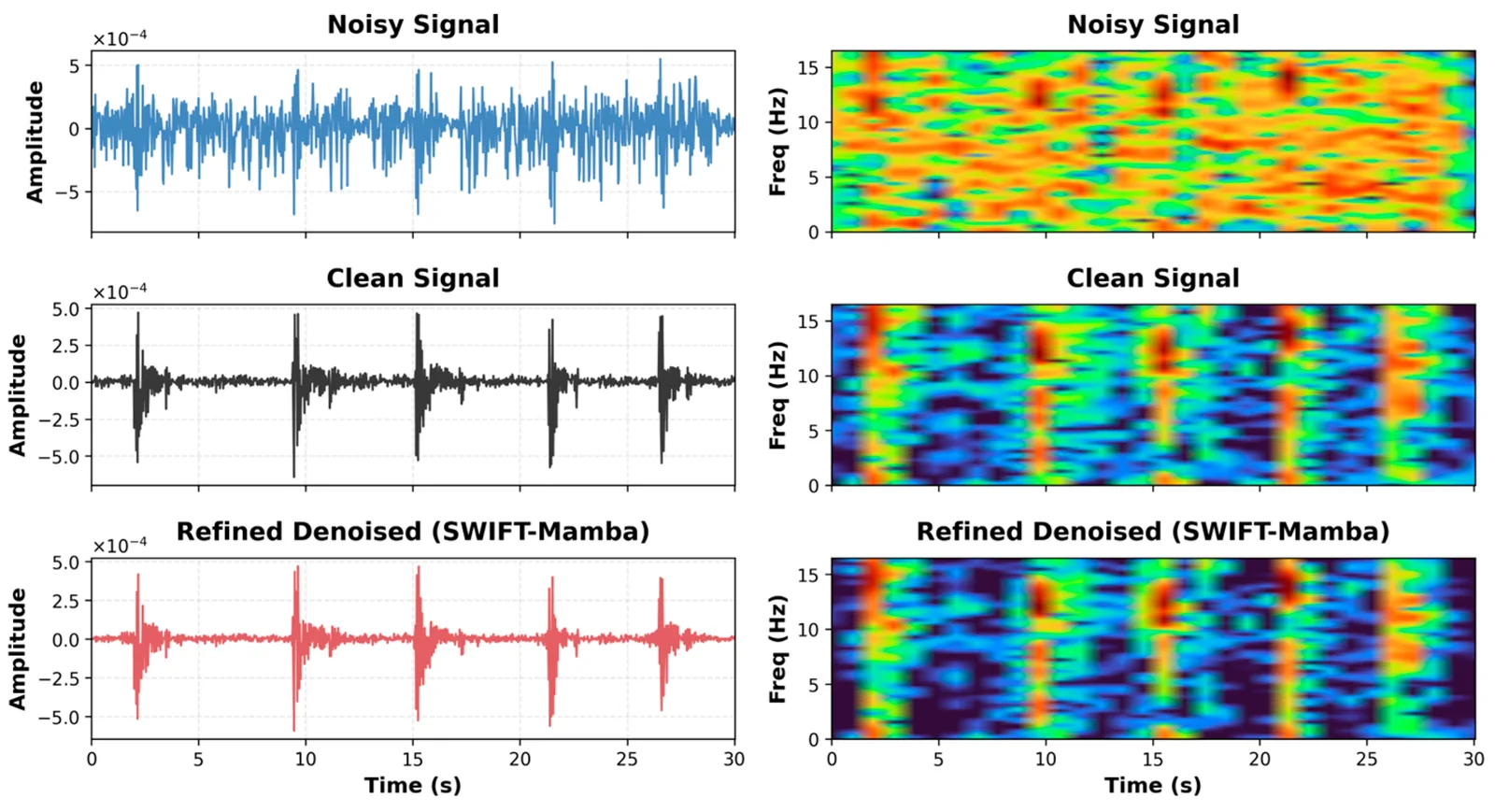
Time-frequency plot of a vibration signal with a frequency of 0.1 Hz and an amplitude of 0.50 mm.

**Figure A2:**
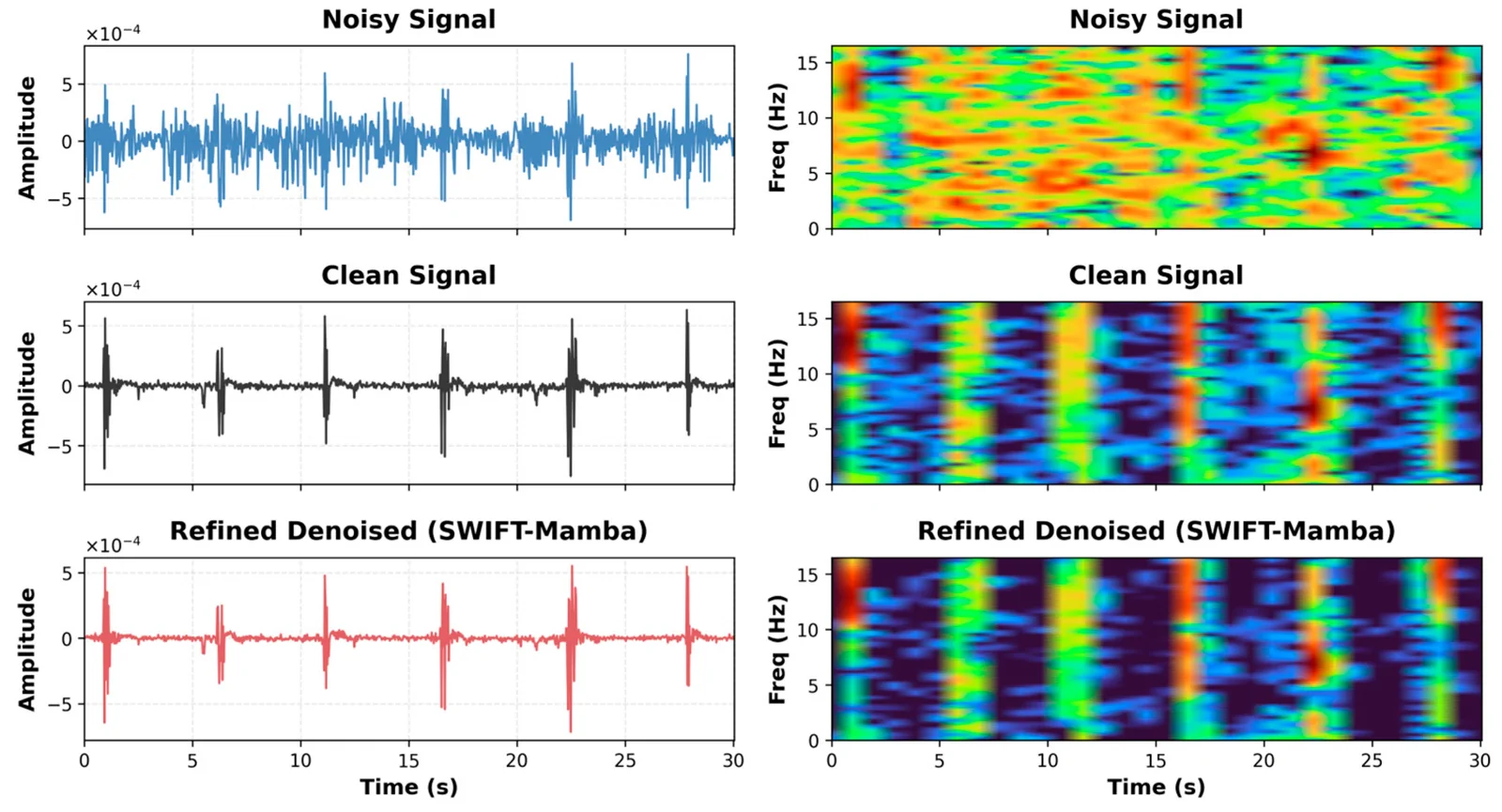
Time-frequency plot of a vibration signal with a frequency of 0.1 Hz and an amplitude of 0.62 mm.

**Figure A3:**
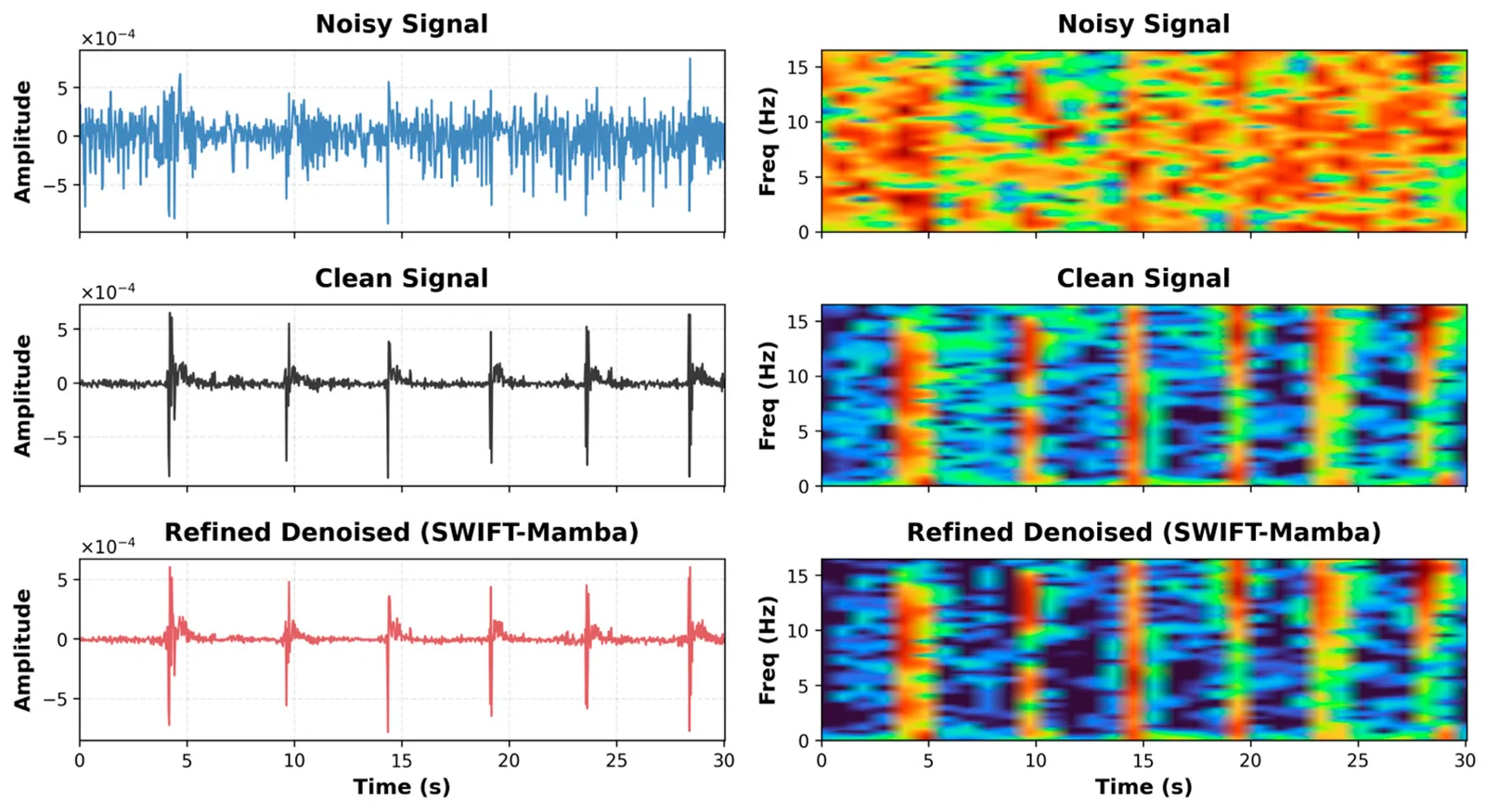
Time-frequency plot of a vibration signal with a frequency of 0.1 Hz and an amplitude of 0.75 mm.

**Figure A4:**
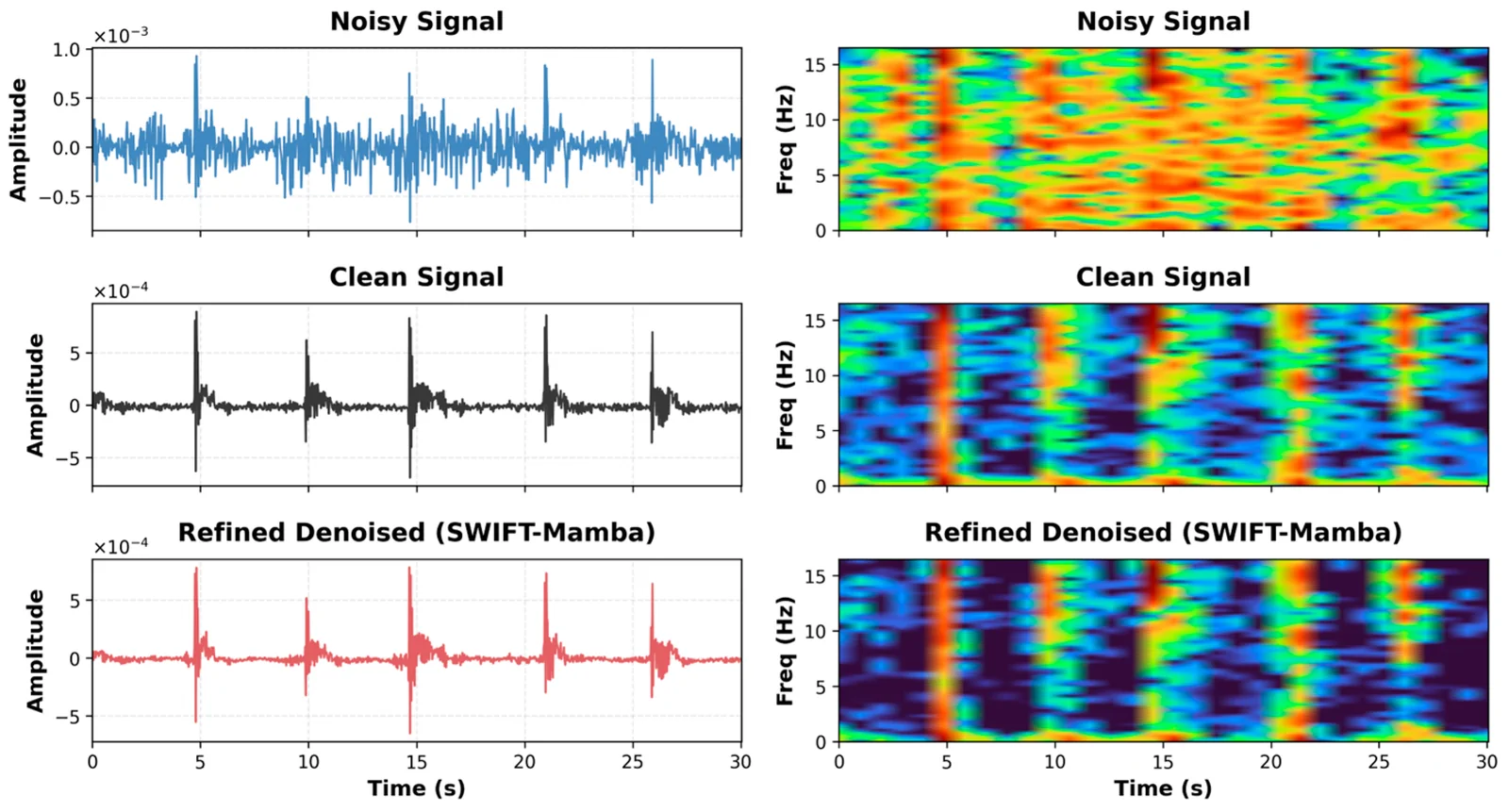
Time-frequency plot of a vibration signal with a frequency of 0.1 Hz and an amplitude of 0.81 mm.

**Figure A5:**
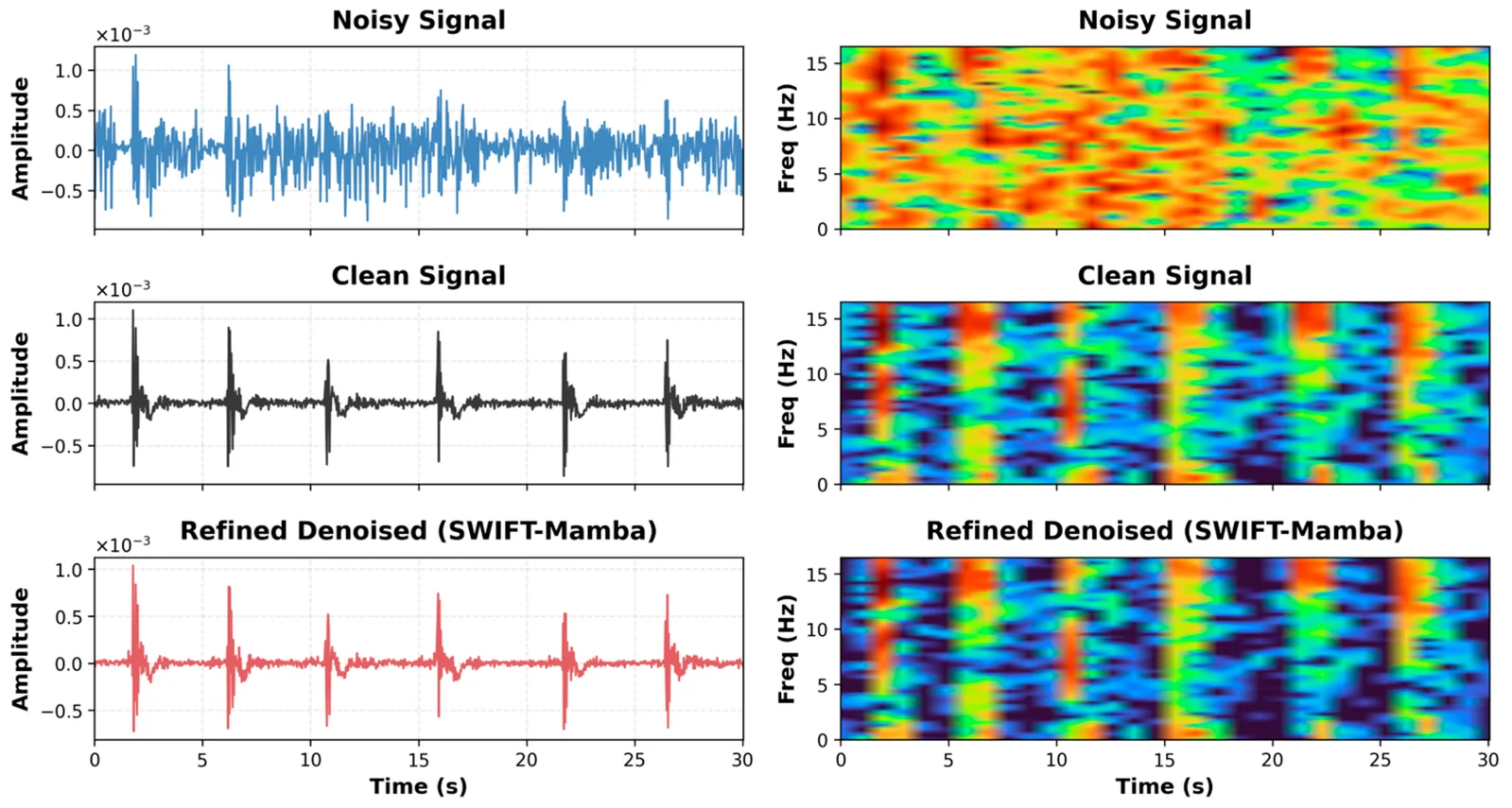
Time-frequency plot of a vibration signal with a frequency of 0.1 Hz and an amplitude of 0.87 mm.

**Figure A6:**
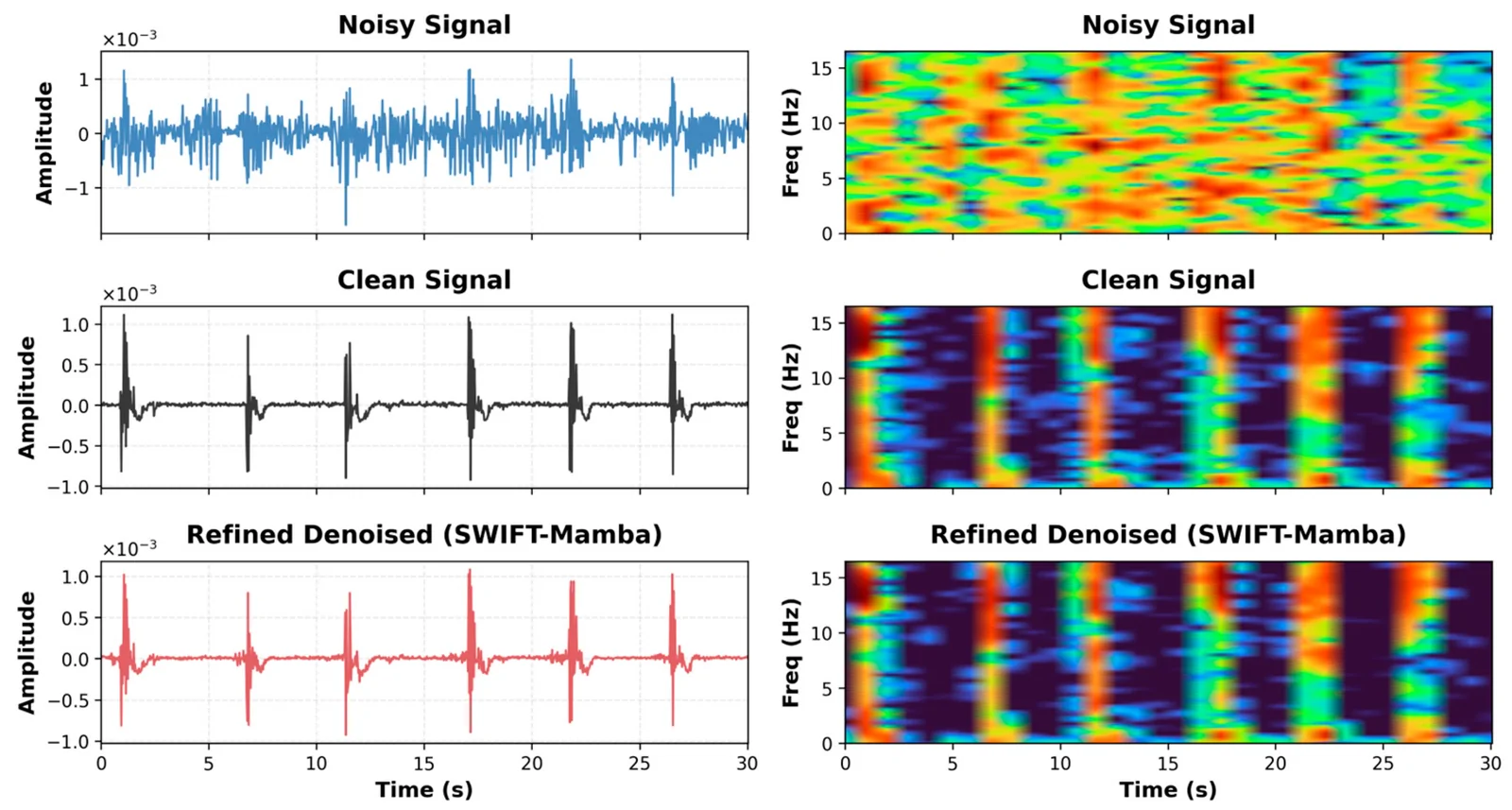
Time-frequency plot of a vibration signal with a frequency of 0.1 Hz and an amplitude of 0.93 mm.

**Figure A7:**
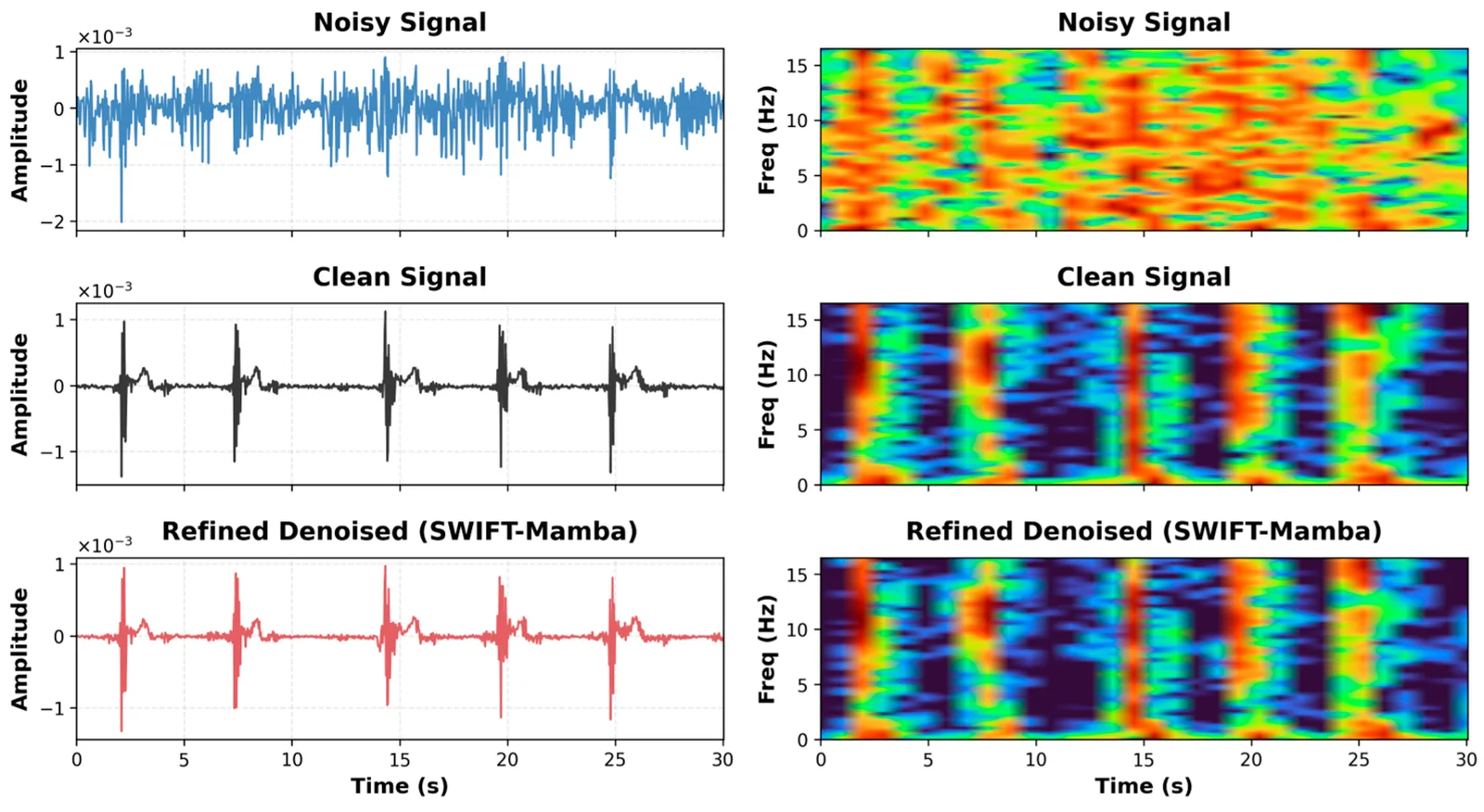
Time-frequency plot of a vibration signal with a frequency of 0.1 Hz and an amplitude of 1.00 mm.

**Figure A8:**
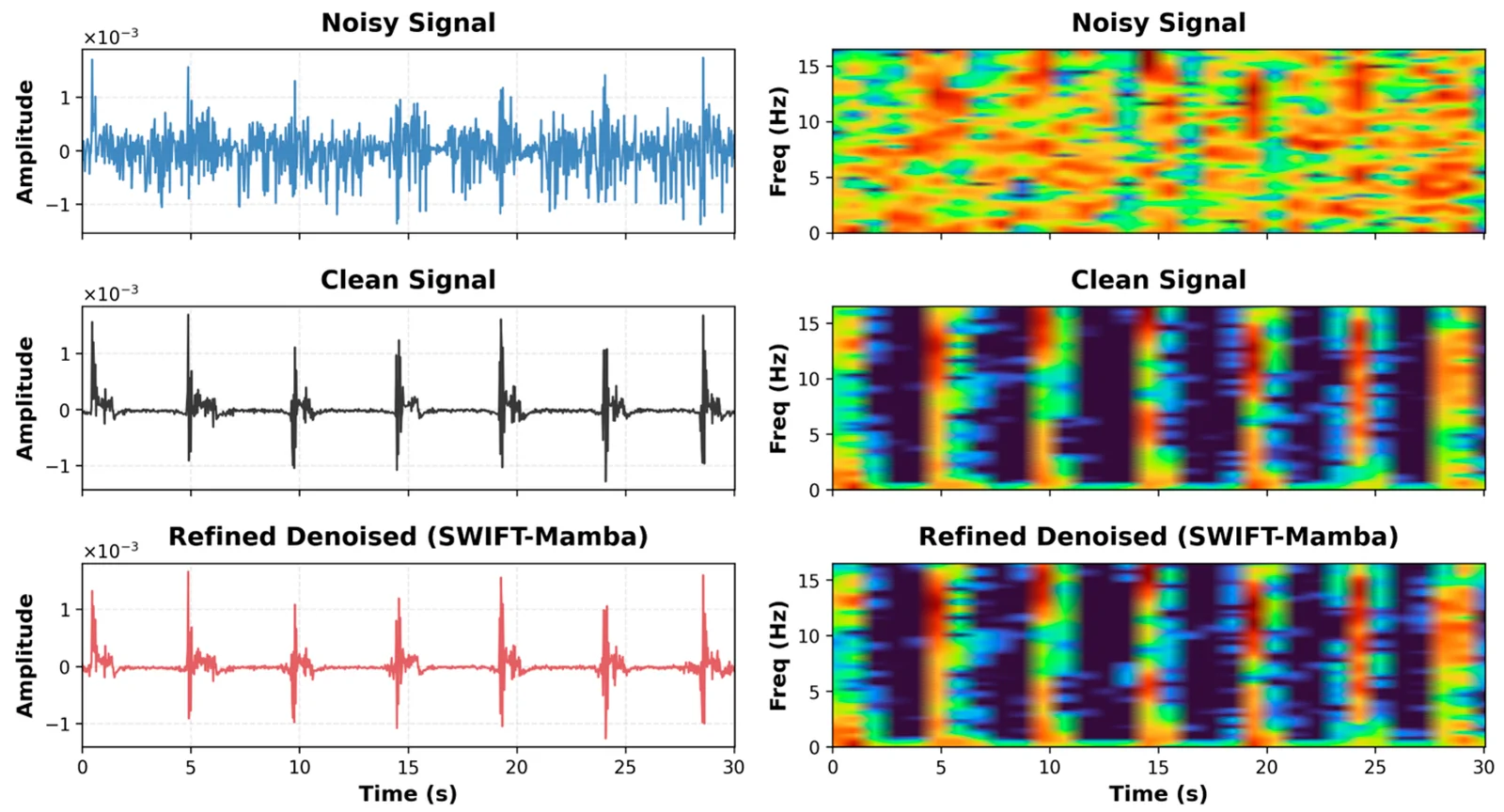
Time-frequency plot of a vibration signal with a frequency of 0.1 Hz and an amplitude of 1.12 mm.

**Figure A9:**
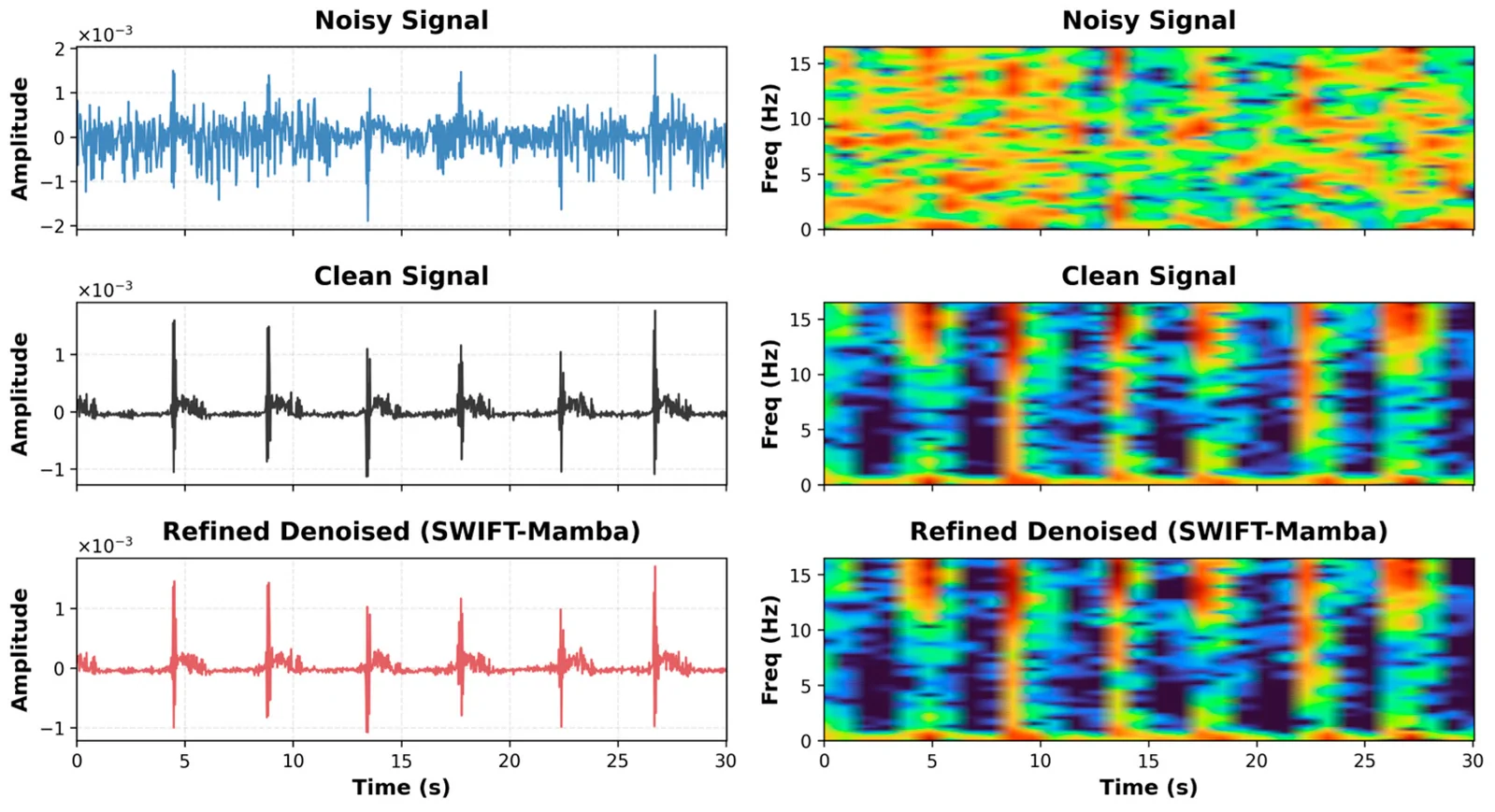
Time-frequency plot of a vibration signal with a frequency of 0.1 Hz and an amplitude of 1.18 mm.

**Figure A10:**
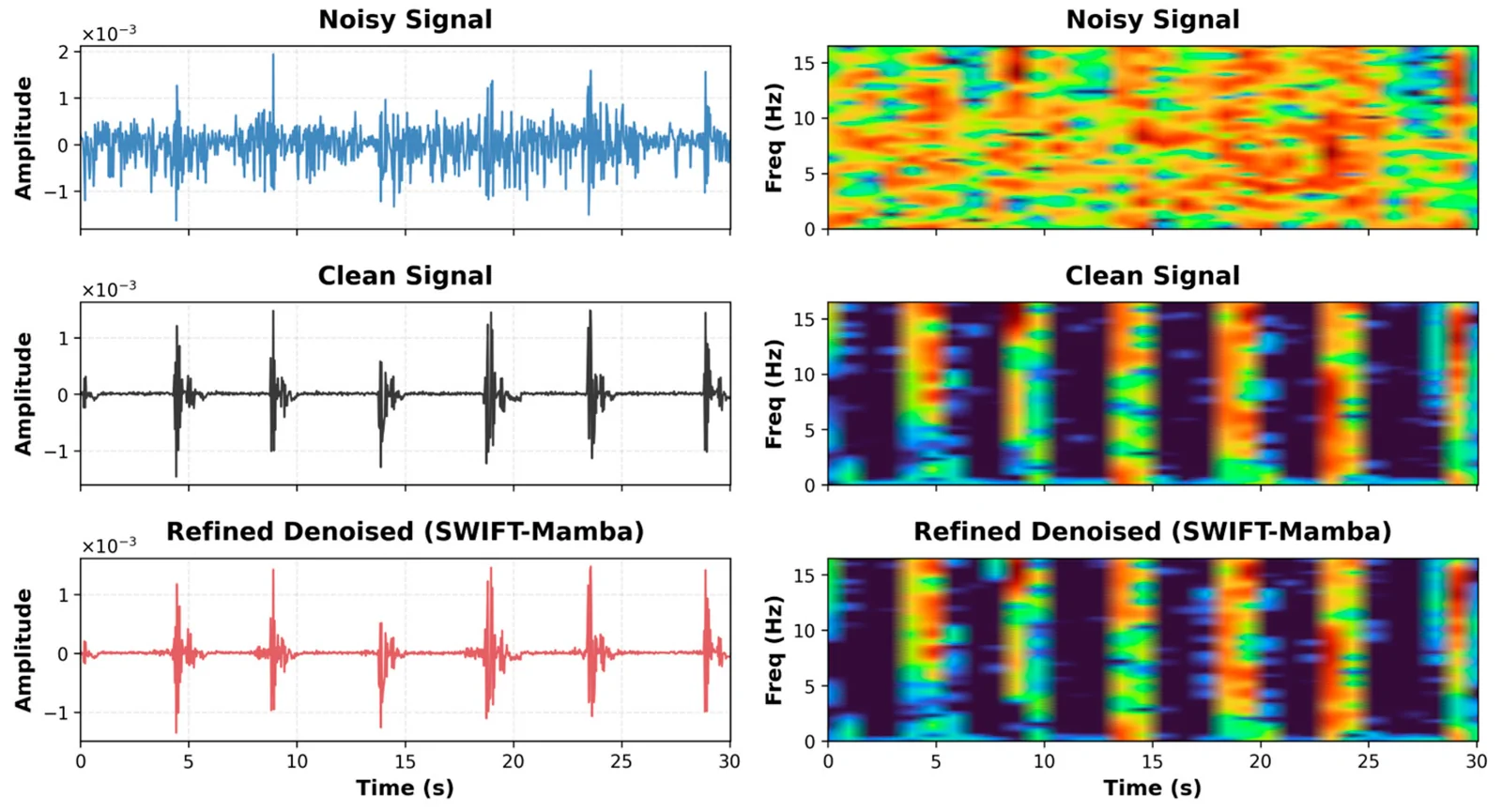
Time-frequency plot of a vibration signal with a frequency of 0.1 Hz and an amplitude of 1.06 mm.

**Figure A11:**
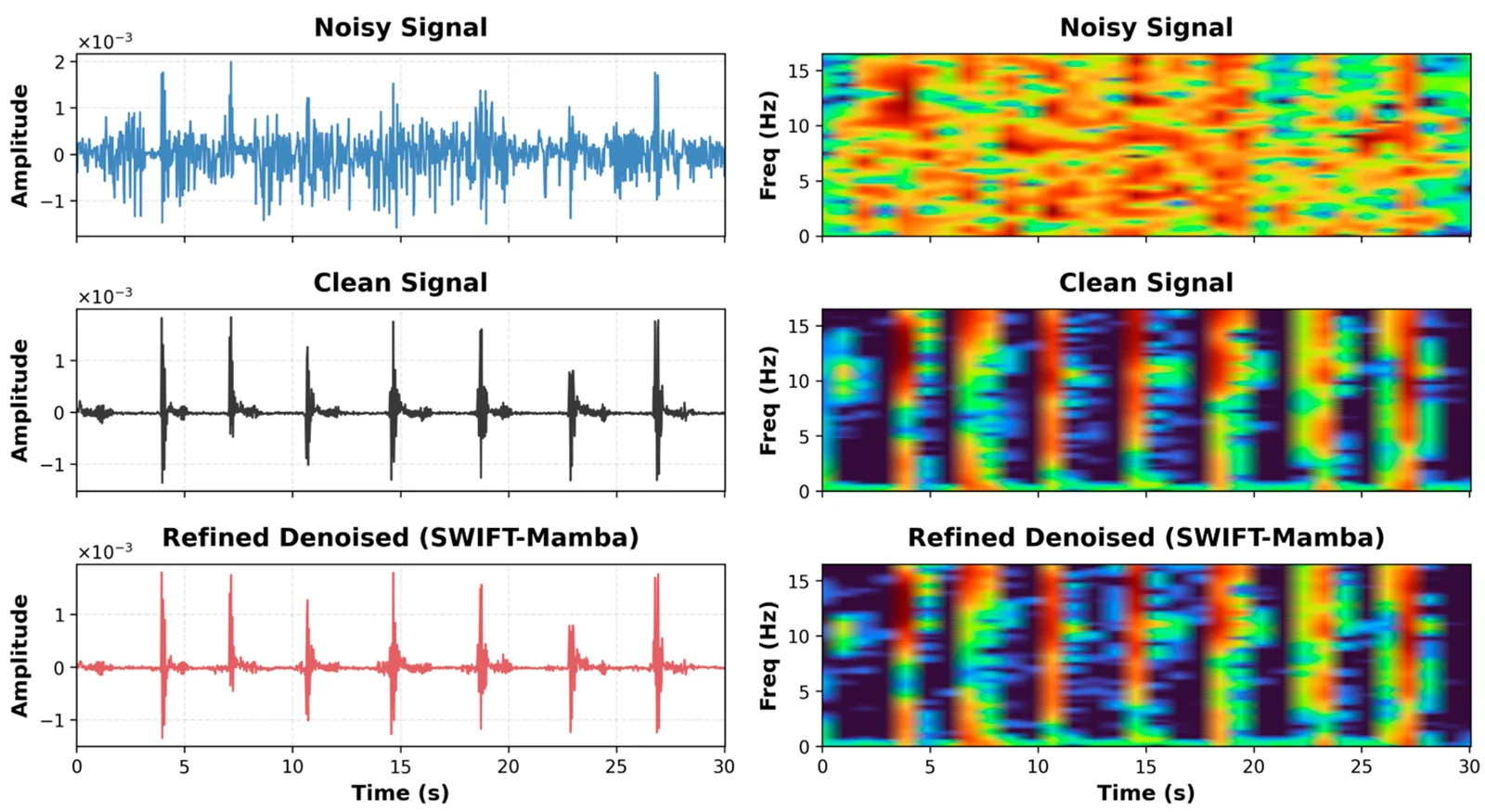
Time-frequency plot of a vibration signal with a frequency of 0.15 Hz and an amplitude of 1.06 mm.

**Figure A12:**
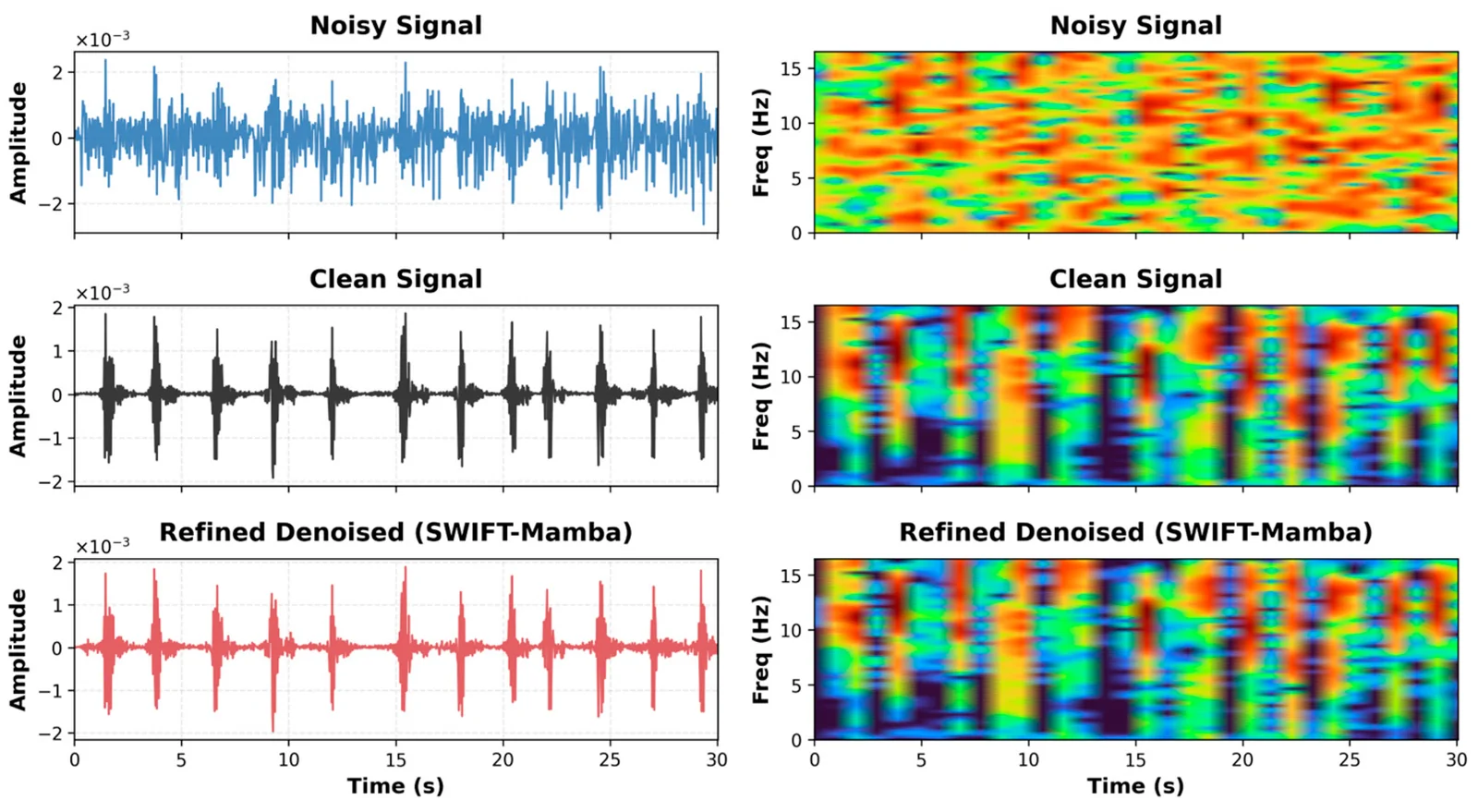
Time-frequency plot of a vibration signal with a frequency of 0.18 Hz and an amplitude of 1.06 mm.

**Figure A13:**
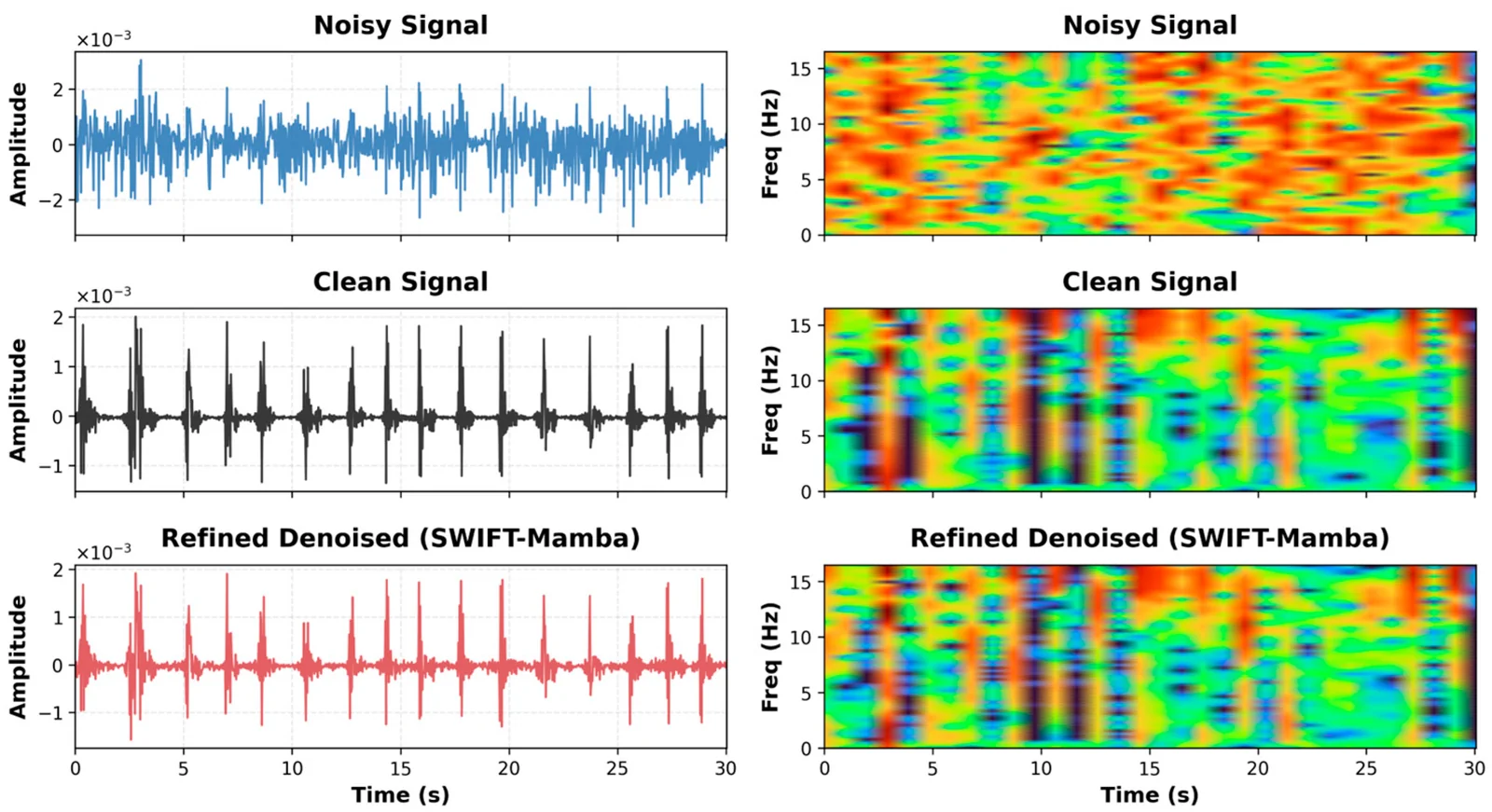
Time-frequency plot of a vibration signal with a frequency of 0.2 Hz and an amplitude of 1.06 mm.

**Figure A14:**
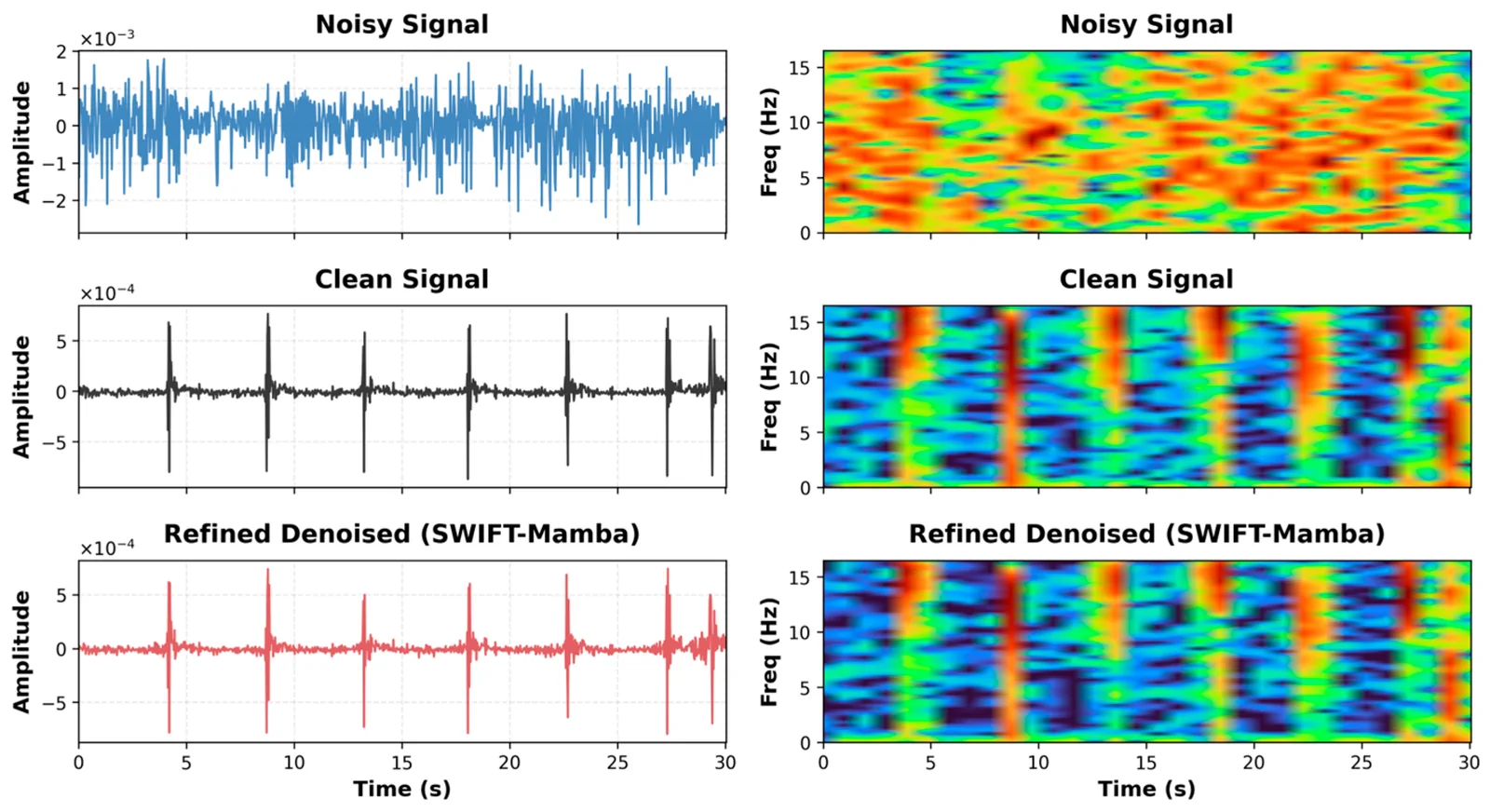
Time-frequency plot of a vibration signal with a signal-to-noise ratio in the range [−20, −15].

**Figure A15:**
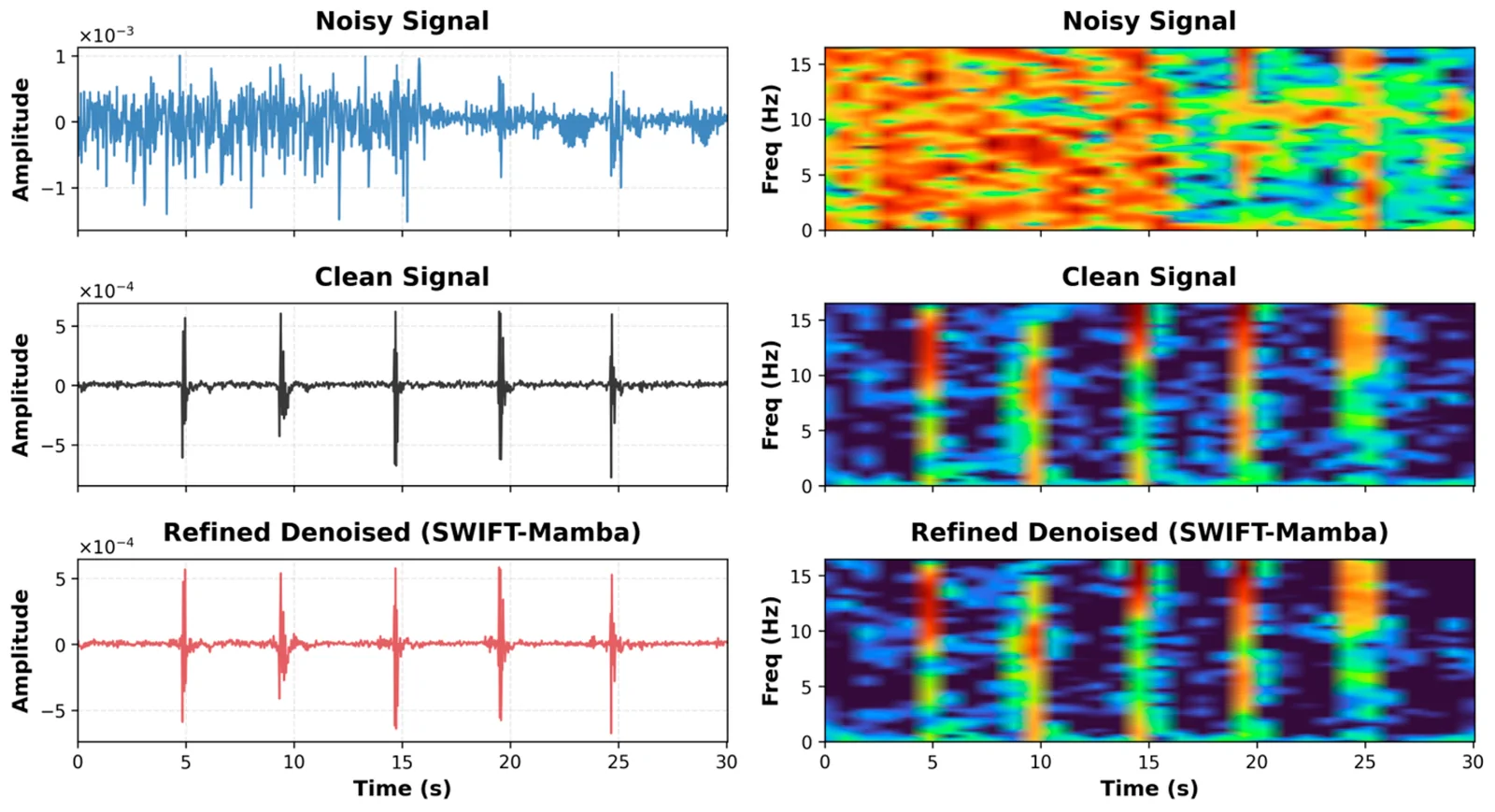
Time-frequency plot of a vibration signal with a signal-to-noise ratio in the range of (−15, −10].

**Figure A16:**
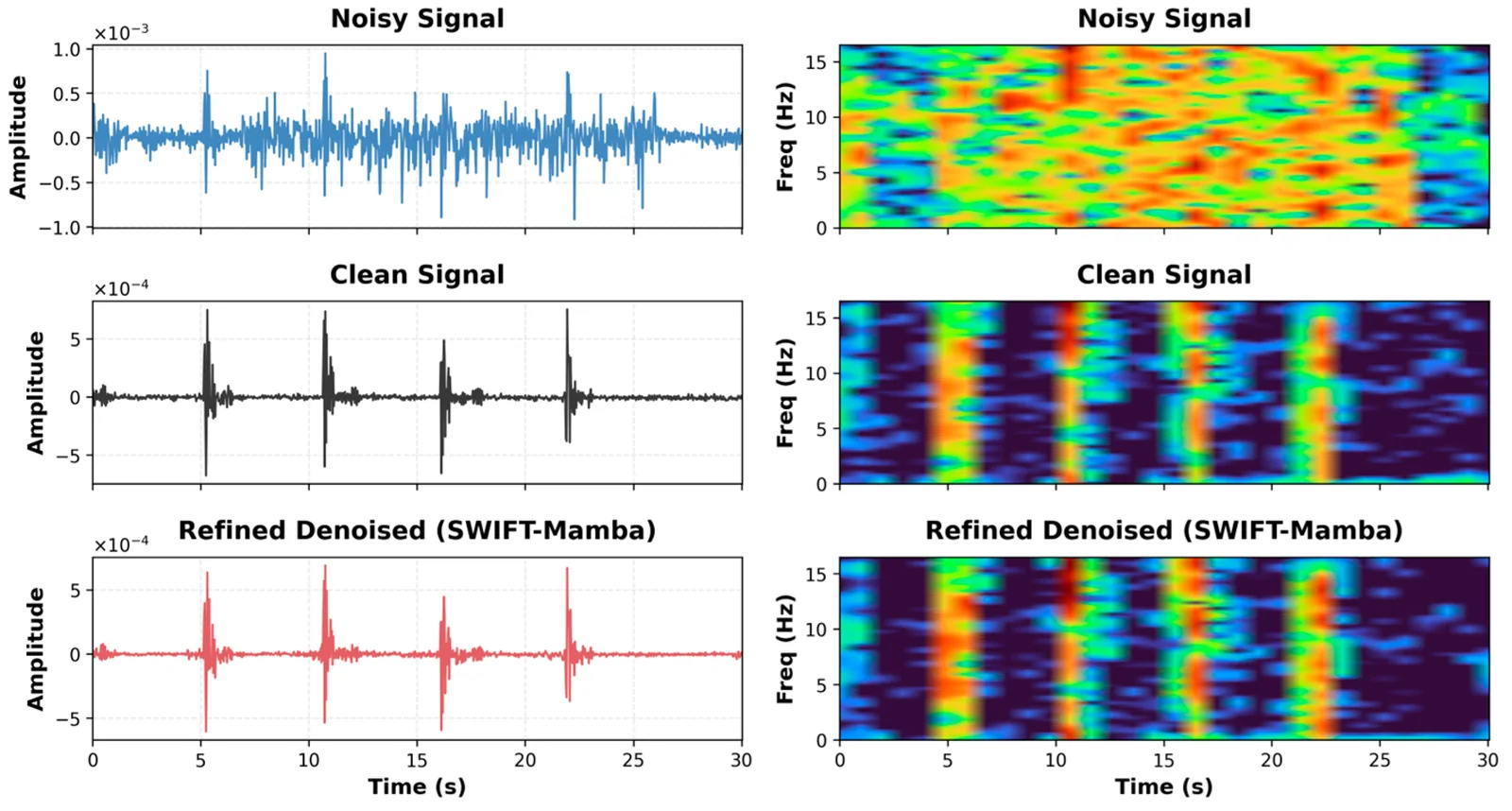
Time-frequency plot of a vibration signal with a signal-to-noise ratio in the range of (−10, −5].

**Figure A17:**
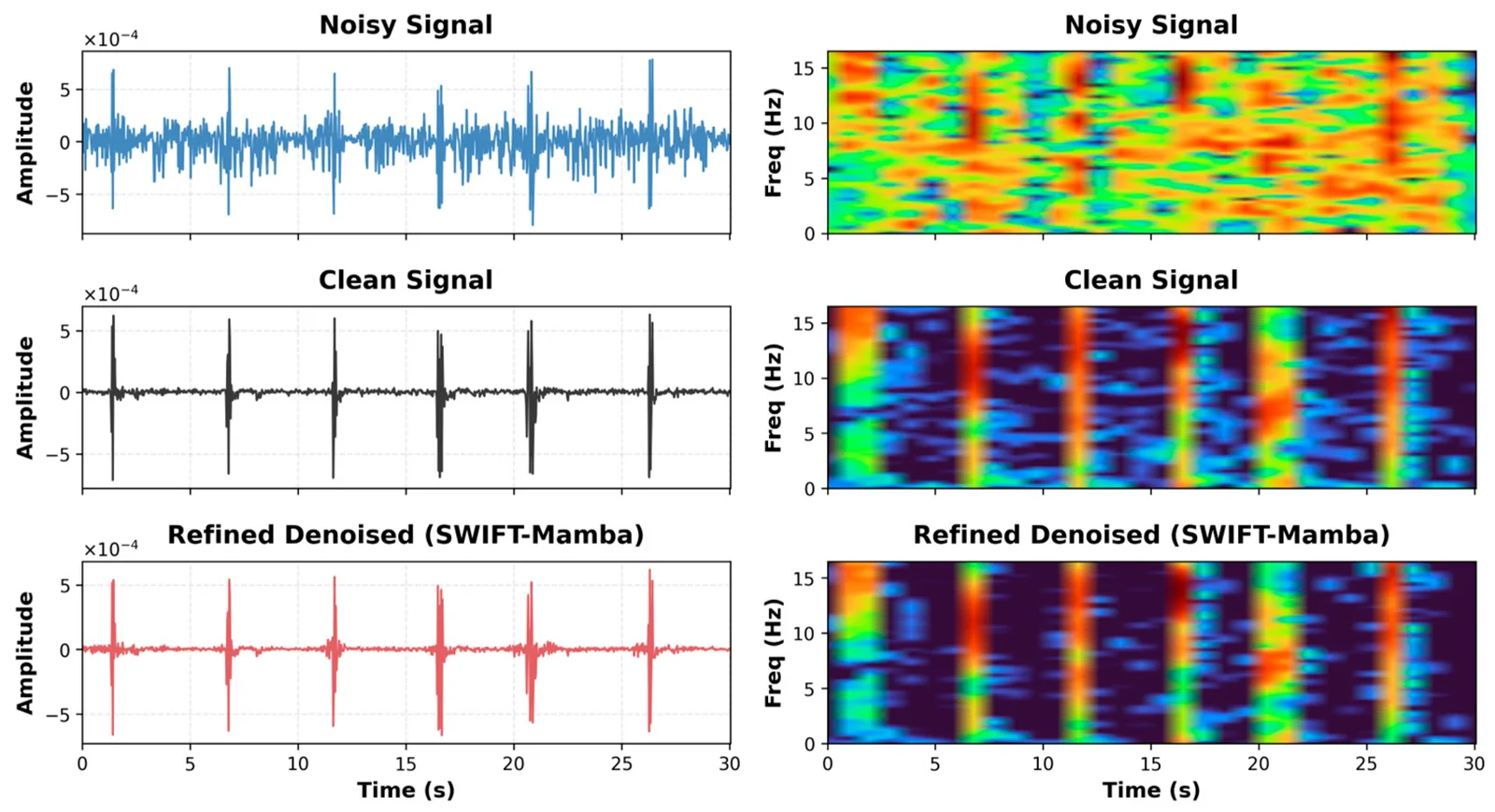
Time-frequency plot of a vibration signal with a signal-to-noise ratio range of (−5, 0).

**Figure A18:**
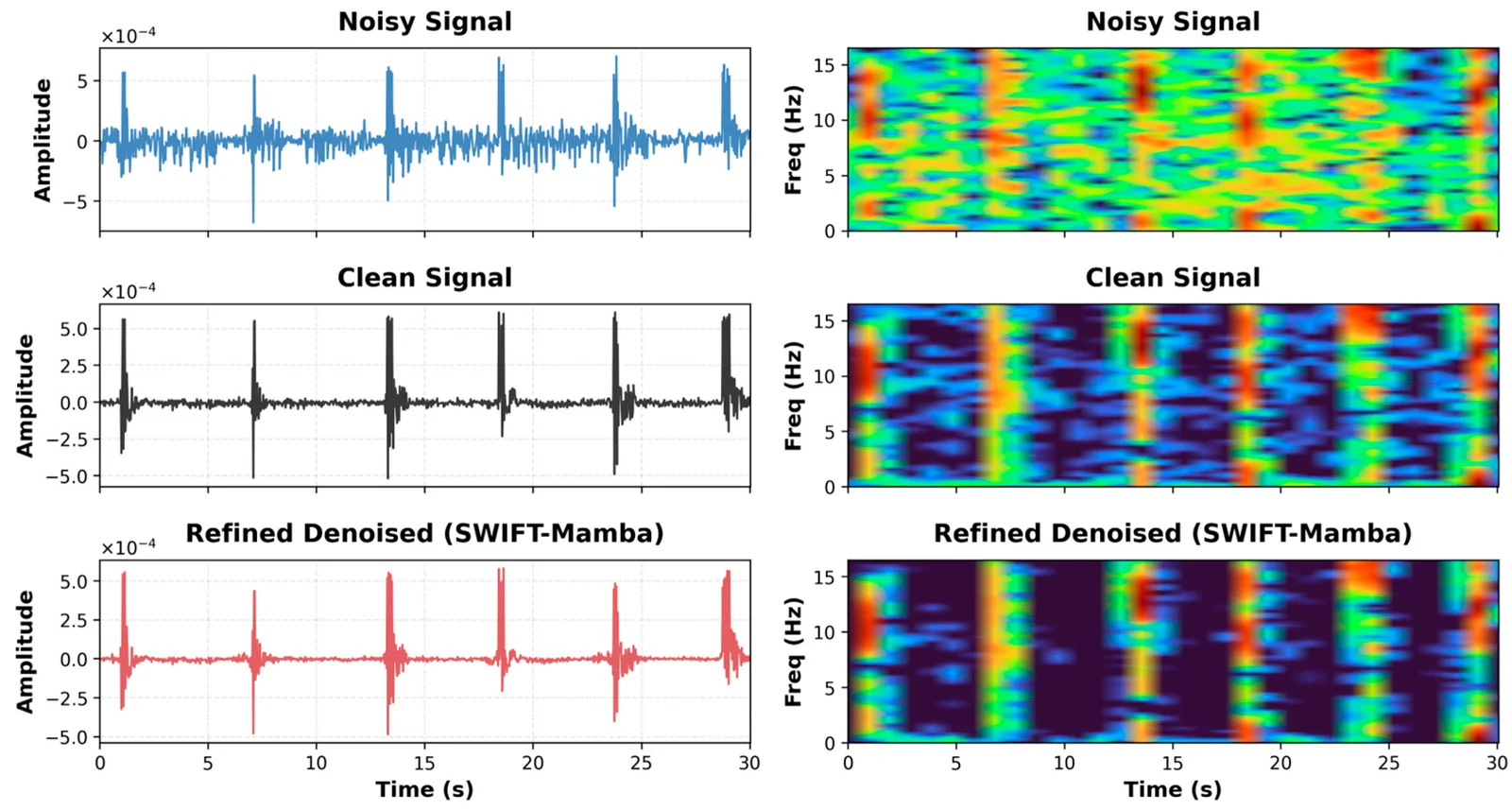
Time-frequency plot of a vibration signal with a signal-to-noise ratio in the range (0, 5].

**Figure A19:**
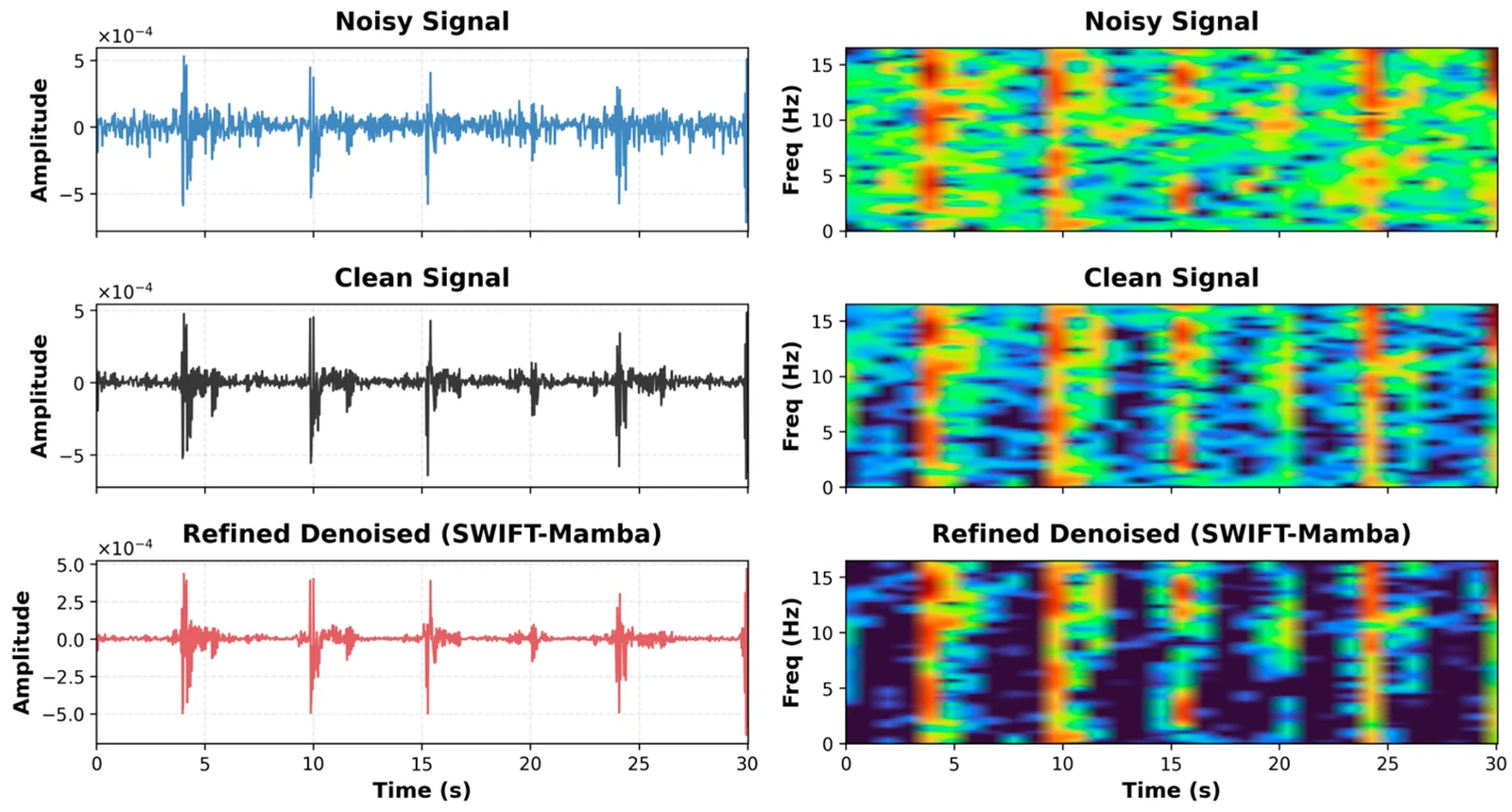
Time-frequency plot of a vibration signal with a signal-to-noise ratio in the range (5, 10].

**Figure A20:**
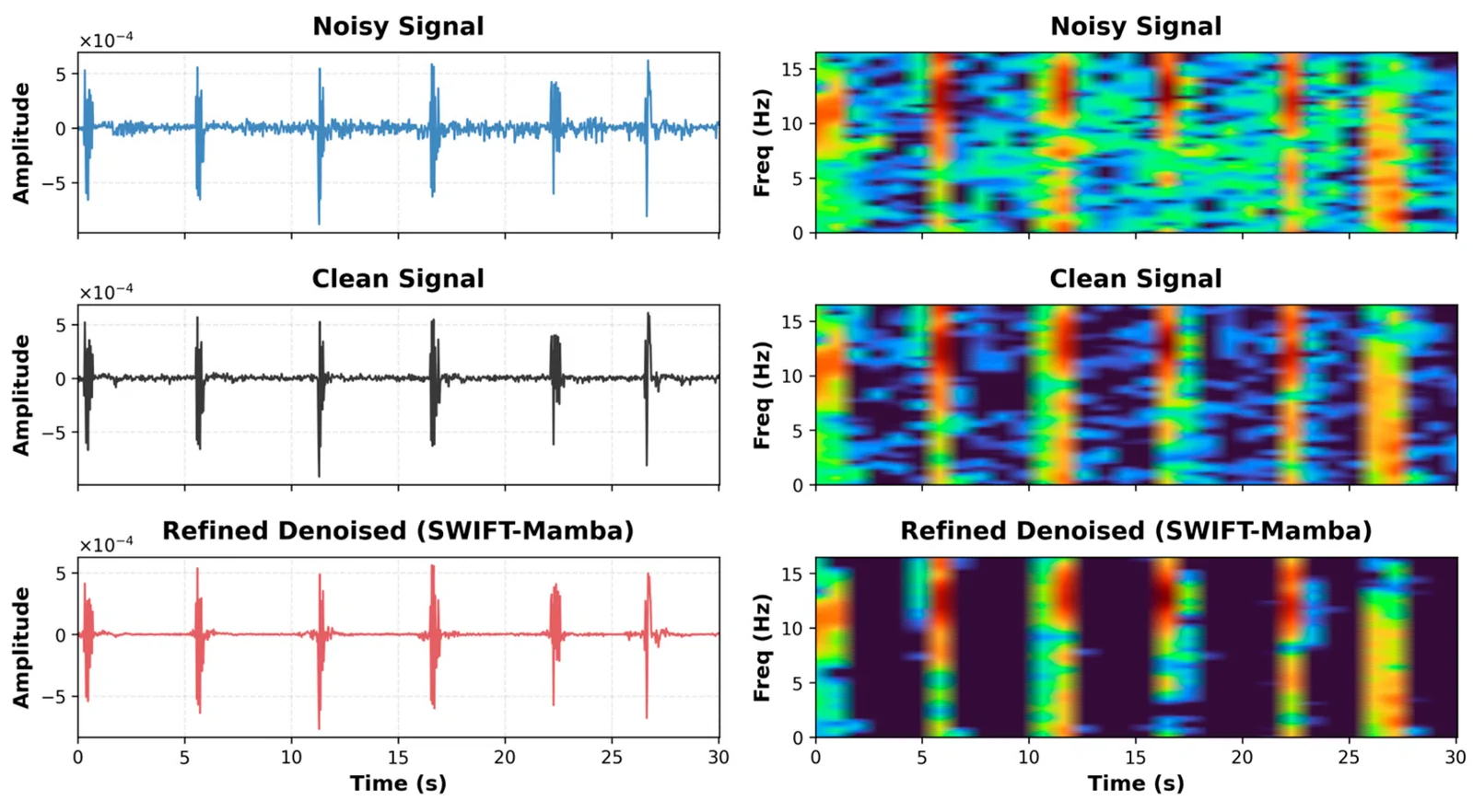
Time-frequency plot of a vibration signal with a signal-to-noise ratio in the range (10–15].

To rigorously evaluate the generalization robustness of SWIFT-Mamba under complex and unknown operating conditions, this section conducts a step-wise stress test on the model using synthetic signals, constructing a wide SNR testing interval spanning from −20 to 15 dB. This span encompasses the entire spectrum of extreme transitional states, ranging from “signals almost completely submerged by non-stationary background noise (negative SNR)” to “near-ideal low-noise conditions (positive SNR).” The performance evolution trends across various intervals are illustrated in Figure 9. Figures showing the noise reduction performance of the synthesized noisy signal under different amplitude and frequency conditions are presented in Appendix A (Figure A1, Figure A2, Figure A3, Figure A4, Figure A5, Figure A6, Figure A7, Figure A8, Figure A9, Figure A10, Figure A11, Figure A12 and Figure A13). As depicted in Appendix B (Figure A14, Figure A15, Figure A16, Figure A17, Figure A18, Figure A19 and Figure A20), the denoised signals maintain the principal waveform morphology of the reference clean signals across different SNR intervals while substantially reducing the noise floor.

1. Maintains good “Safety-Net” Capability under Extremely Degraded Conditions (Background Suppression Priority)

Most strikingly, within the extreme high-noise interval of −20 to −15 dB on the left side of the charts (i.e., conditions where the authentic wavefront signal is almost entirely overwhelmed by background clutter), the metrics of the input signal (blue line) exhibit severe degradation. However, the reconstructed output of SWIFT-Mamba (highlighted red line) demonstrates a highly robust safety-net capability, the model reverses the trend, elevating the sub −15 dB input SNR to 13.82 dB (Figure 9a), achieving an absolute net gain of nearly 29 dB. Even more remarkably, regarding the NMSE metric (Figure 9d), logarithmic axis), which reflects the degree of waveform distortion, the model compresses a disastrous original error of up to 32.8 by nearly three orders of magnitude to approximately 0.018. This demonstrates that, owing to the powerful global receptive field of the 1D-SSM, the network can accurately strip out the underlying base manifold of the wavefront signal even at extremely low SNRs, thereby averting the direct failure typically experienced by traditional shallow networks when confronted with intense noise.

2. Ultimate “Fidelity” Capability under High-SNR Conditions (Phase Topology Priority)

As the input SNR progressively improves (from the 0 to 15 dB interval), background noise gradually diminishes. At this stage, overly aggressive denoising is highly prone to destroying the high-frequency features of the wavefront signal. As observed in the right-hand intervals of the charts, SWIFT-Mamba intelligently accomplishes a smooth strategic transition. Given that the input signal already possesses high quality, the model’s output red line still firmly suppresses the input blue line, averting any metric inversion. Its Pearson PCC closely hugs an extremely high level of 98.8% (Figure 9e), while the LSD stabilizes at a remarkably low level of 0.06 to 0.07 dB (Figure 9f). This also confirms that the physics-based Soft-Clipping mechanism proposed in this paper has certain advantages, excising residual faint clutter while fundamentally introducing no secondary phase distortion caused by network over-smoothing.

In short, whether dealing with strong noise capable of disrupting traditional algorithms or subtle noise that could cause high-frequency topological distortions, SWIFT-Mamba maintains exceptionally robust “Positive Net Gain” across broadband fluctuation tests spanning up to 35 dB. This adaptability and robustness under extreme physical boundary conditions effectively reduce the risk of massive data invalidation caused by sporadic strong gust interference in drone-based in situ verification systems.

### 5.2. Comprehensive Comparative Experiments

**Figure 10:**
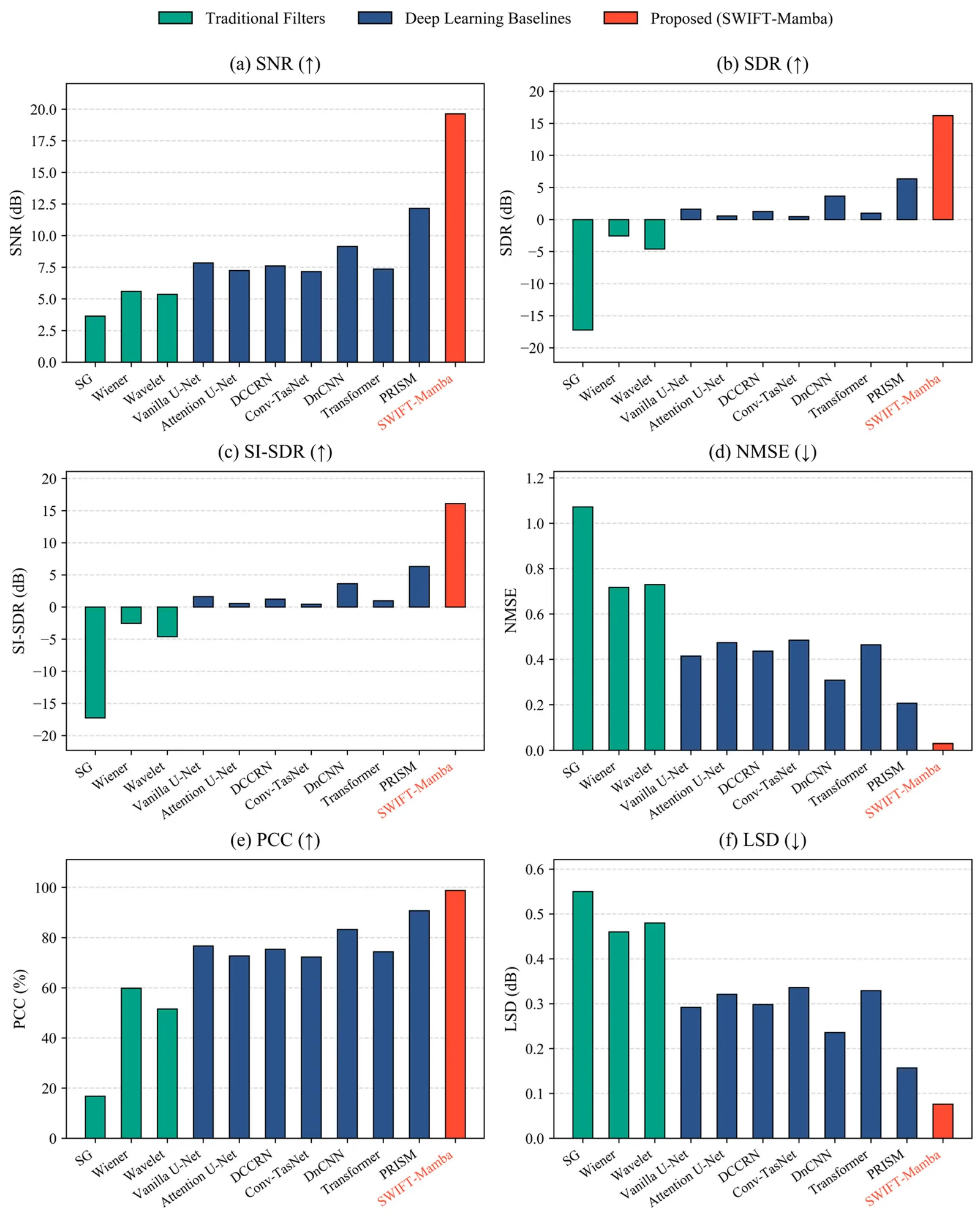
Comparative performance of different denoising methods on laboratory-measured noisy signals. The six subplots report SNR, SDR, SI-SDR, NMSE, PCC, and LSD, respectively. ↑ indicates that a higher value is better, while ↓ indicates that a lower value is better. The SNR, SDR, and SI-SDR values shown in the figure are all gain values. The NMSE, PCC, and LSD values are all output values.

**Figure 11:**
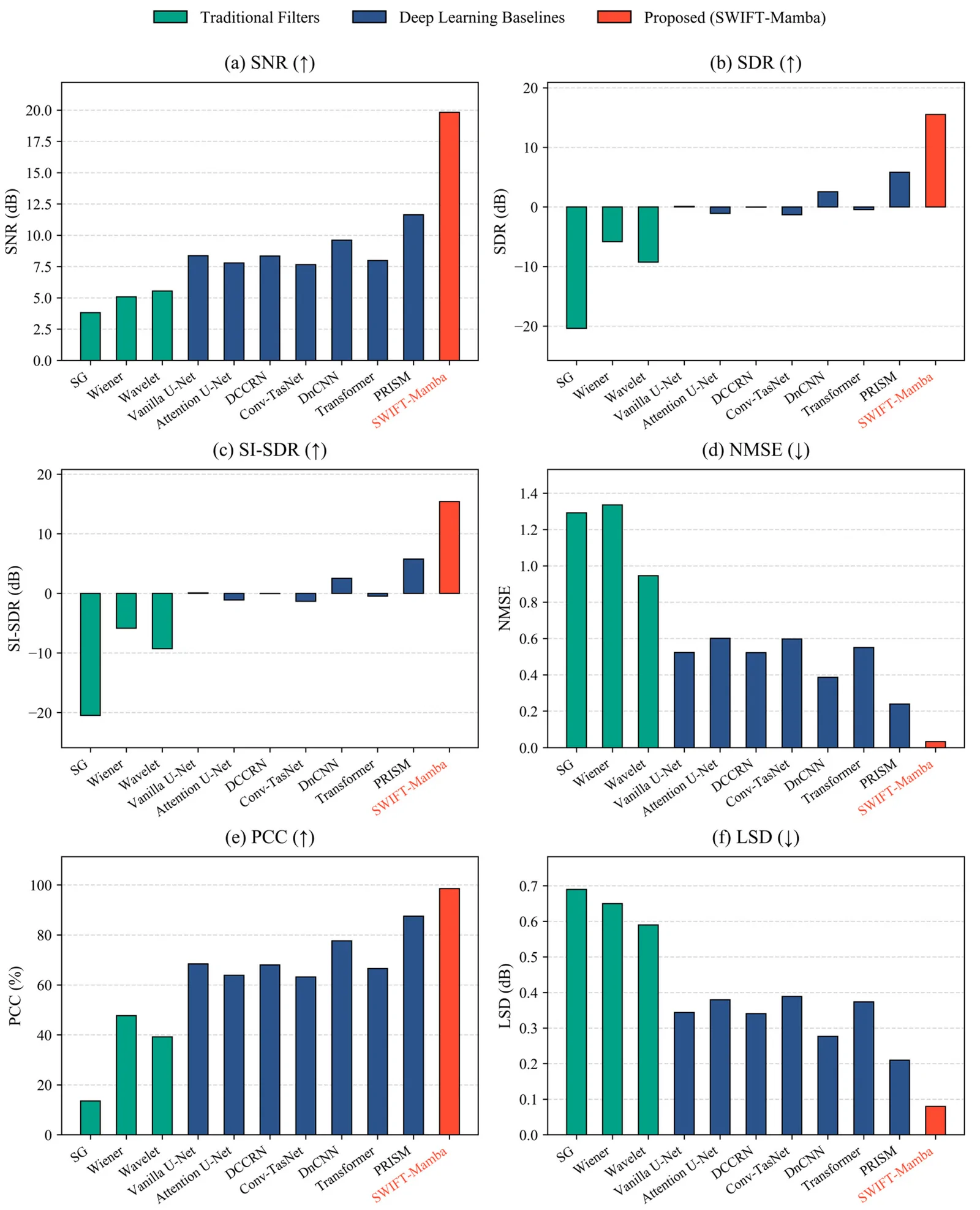
Comparative performance of different denoising methods on synthetic noisy signals across different SNR conditions. The six subplots report SNR, SDR, SI-SDR, NMSE, PCC, and LSD, respectively. ↑ indicates that a higher value is better, while ↓ indicates that a lower value is better. The SNR, SDR, and SI-SDR values shown in the figure are all gain values. The NMSE, PCC, and LSD values are all output values.

To systematically evaluate the effectiveness of the proposed SWIFT-Mamba model in the wavefront slope signal cleaning task, this section conducts a comprehensive comparison between our method and 10 representative baseline algorithms on both laboratory-measured signals and synthetic signals. The baselines encompass three traditional digital filtering algorithms (SG filter, Wiener filter, and Wavelet denoising) and seven cutting-edge deep learning architectures (including CNN-based Vanilla U-Net, Attention U-Net, DnCNN; RNN-based DCCRN, Conv-TasNet; Self-attention-based Transformer; and the complex domain SOTA model PRISM). To ensure a fair comparison, all evaluated baseline models were trained, tuned, and tested under strictly identical experimental conditions and data splits. The intuitive performance comparison bar charts for all core metrics correspond to Figure 10 and Figure 11, respectively.

#### 5.2.1. Generalization Analysis on the Laboratory-Measured Dataset

To bridge the sim-to-real gap inherent in deep learning models and to verify the inverse generalization capability of the SWIFT-Mamba algorithm under real-world physical operating conditions absent from the training phase, this section first conducts a rigorous blind denoising evaluation on a noisy dataset authentically collected from a physical laboratory platform. Compared to ideally distributed synthetic signals, the measured wavefront signals are deeply coupled with non-stationary interferences that are exceedingly difficult to accurately model mathematically, such as high-frequency mechanical vibrations and complex air turbulence perturbations. These highly stochastic, out-of-distribution (OOD) noise components not only severely corrupt the intrinsic topological structure of the target signals but also impose extremely stringent demands on the dynamic robustness and boundary-perception capabilities of the model in actual industrial deployment. The detailed results of the time-domain waveform recovery and spectral feature reconstruction are illustrated in Figure 10.

As shown in Figure 10, traditional digital filtering algorithms (such as SG filtering, Wiener filtering, and wavelet denoising) exhibit the poorest performance across all evaluated metrics. It can be intuitively observed from Figure 10b,c that both the SDR and SI-SDR of these methods present severe negative collapses, while Figure 10d,f reveal exceptionally high NMSE and LSD errors. This corroborates that traditional algorithms heavily rely on strict mathematical priors and stationarity assumptions, and can only perform noise separation through local time-domain smoothing or predefined frequency-domain basis functions. Lacking learnable adaptive parameters, these methods typically struggle to dynamically track the complex variations in high-frequency transient signals when encountering unknown, non-stationary broadband interferences in real-world measured environments.

On the other hand, the cluster of deep learning baseline models demonstrates a step-wise performance leap in the charts, verifying the advantages of data-driven approaches: CNN-based architectures (e.g., Vanilla U-Net and DnCNN) excel at capturing time-frequency mapping relationships within local receptive fields; RNN-based architectures (e.g., DCCRN and Conv-TasNet) focus on modeling temporal dependencies; whereas Transformer and PRISM introduce global attention mechanisms and complex domain feature processing, respectively, achieving sub-optimal performance in the baseline comparison. However, observing the overall height of the bar charts reveals that the performance growth of these heavy networks has encountered a prominent bottleneck when processing blind data. This indicates their over-reliance on the data distribution of the training set. When generalizing to blind environments containing real physical perturbations, their inherent architectures are highly prone to robustness challenges such as amplitude overfitting or phase truncation.

In contrast, as shown by the prominent red bar chart in the figure, SWIFT-Mamba alleviates the aforementioned performance bottleneck and demonstrates strong performance across all six metrics. Specifically, Figure 10a shows that SWIFT-Mamba achieves a significant improvement in signal-to-noise ratio (SNR) on the test dataset, reaching 19.64 dB, which maintains a substantial performance lead of 7.48 dB over the sub-optimal baseline (PRISM, 12.16 dB). More notably, Figure 10e demonstrates that the Pearson correlation coefficient (PCC) of the proposed model approaches the broad-scale boundaries, reaching 98.81%. This implies a high degree of consistency between the physical envelope of the reconstructed signal and the ground-truth pure wavefront signal. Meanwhile, its SDR (Figure 10b) stabilizes at a high level of 16.21 dB, whereas the error metrics (NMSE and LSD) in Figure 10d,f are extremely compressed to a minimal baseline almost touching the horizontal axis. This cross-dimensional, comprehensive leading performance under measured environments preliminarily verifies the effectiveness of the proposed Soft-Clipping complex mask mechanism; when dealing with real and extreme transient sudden peaks, it effectively mitigates the phenomena of phase truncation and amplitude overfitting that frequently occur in conventional networks.

#### 5.2.2. Performance Analysis on the Synthetic Signals

The preceding blind denoising experiments have fully corroborated the exceptional generalization robustness of SWIFT-Mamba against unknown, non-stationary interferences in real-world physical environments. However, to effectively isolate the statistical uncertainties introduced by unmeasurable perturbations (e.g., sudden mechanical vibrations and uncontrollable airflows) in actual measured scenarios, and to further quantitatively investigate the Theoretical Denoising Upper Bound of the algorithm under ideal boundary conditions, this section supplements a performance analysis based on a strictly controlled synthetic dataset. In contrast to the measured blind data, this synthetic dataset constructs a benchmark sample library covering multiple distinct signal-to-noise ratio (SNR) gradients by strictly mixing pure baseline wavefront signals with precisely mathematically modeled distribution noise in specific proportions. This aims to test and compare the feature extraction precision and absolute waveform reconstruction fidelity of each model at a purely mathematical mapping level. The detailed comparisons of multi-dimensional quantitative evaluation metrics among various mainstream baseline algorithms and the proposed model on this synthetic benchmark are illustrated in Figure 11.

As illustrated by the bar chart distribution in Figure 11, traditional filtering algorithms exhibit distinct limitations when addressing such non-stationary broadband noise. It can be intuitively observed from Figure 11c that, taking SG filtering and Wavelet as examples, their SI-SDR gain metrics plummet to −20.47 dB and −9.28 dB, respectively, exhibiting a severe negative collapse. This indicates that traditional methods based on linearity and stationarity assumptions induce severe physical phase distortion and signal corruption while filtering out complex background noise.

On the other hand, the cluster of deep learning baseline models demonstrates fundamental noise suppression capabilities; however, when confronted with the extremely large signal-to-noise ratio (SNR) span in the synthetic signals, the robustness challenges of their inherent architectures are thoroughly exposed. It is clearly visible in the charts that mainstream real-domain mapping networks (e.g., Vanilla U-Net and Transformer) exhibit limited physical fidelity, with their SI-SDRs generally falling below 1 dB. More notably, Figure 11b,c reveal that for models represented by Attention U-Net, DCCRN, and Conv-TasNet, their SDR and SI-SDR bars even extend inversely into the negative axis (e.g., the SI-SDR of Conv-TasNet is −1.31 dB). In a physical sense, this implies that the networks not only fail to filter out the noise effectively but, owing to gradient oscillations and uncontrolled mask estimation, introduce additional nonlinear distortions, thereby severely disrupting the topological structure of the original signal. Leveraging its complex domain processing mechanism, the PRISM model achieves an SNR gain of 11.64 dB and an SI-SDR gain of 5.78 dB among the baseline networks. However, as observed from Figure 11a,e, its denoising upper bound (e.g., the PCC stagnates at 87.51%) remains hindered.

In contrast, as shown by the prominent red bar chart in the figure, the proposed SWIFT-Mamba demonstrates superior performance under such strictly controlled conditions. Figure 11a,c show that SWIFT-Mamba achieves the best performance among all compared methods. Its SNR gain reaches 19.82 dB, exceeding PRISM by 8.18 dB, while its SI-SDR gain reaches 15.42 dB. Additionally, its SDR gain stabilizes at 15.54 dB, and the PCC approaches the broad-scale boundaries, reaching 98.55% (Figure 11e). Simultaneously, Figure 11d,f demonstrate that the LSD metric, which characterizes the preservation capability of high-frequency transient details, is effectively compressed to 0.080 dB, and the NMSE drops to 0.0345, with the red bars practically touching the horizontal axis baseline. This fully demonstrates the adaptive regulation capability of the proposed complex domain dynamic Soft-Clipping mechanism, effectively preventing the model from falling into the trap of over-smoothing under extreme conditions such as minor noise interference or severe noise coverage.

Synthesizing the evaluation results from both the blind denoising and synthetic datasets, it can be concluded that, attributed to the synergistic effect of the time-frequency dual-track architecture and the 1D-SSM hardware-aware scanning, SWIFT-Mamba accurately extracts and decouples the nonlinear manifold features of the time series without losing high-frequency harmonics in the frequency domain. While maintaining lightweight inference suitable for edge deployment (as discussed in Section 5.3), this model establishes distinct advantages over existing methods in terms of reconstruction accuracy, physical phase fidelity, and generalization in real-world scenarios. Consequently, it provides a highly viable algorithmic solution for the high-quality cleaning of UAV airborne wavefront signals.

### 5.3. Comparative Analysis of Computational Complexity and Lightweight Design

To comprehensively evaluate the overall deployment potential of the models in edge computing scenarios, this section conducts a systematic comparative analysis among SWIFT-Mamba, cutting-edge deep learning baseline models, and classic traditional filtering algorithms from two dimensions: trade-off analysis between edge-device inference latency and reconstruction accuracy, and theoretical computational complexity evaluation of deep learning models.

#### 5.3.1. Theoretical Computational Complexity Evaluation of Deep Learning Models

A model’s storage footprint on memory-constrained devices, along with its theoretical computational power consumption, are key factors in determining whether it can be deployed at the edge. High storage requirements (directly determined by the number of parameters) can quickly deplete limited static random-access memory (SRAM), while high computational overhead (quantified in FLOPs) can directly cause the processor to enter a high-power, high-heat state, triggering thermal throttling and affecting data processing rates.

In this subsection, to systematically evaluate the resource compatibility of the algorithm proposed in this paper in an edge computing environment, we compare the theoretical parameter counts (Params) and floating-point operation requirements (FLOPs) of SWIFT-Mamba with those of various mainstream deep learning denoising architectures (covering a range of cutting-edge, resource-intensive models based on traditional CNNs, RNNs, and Transformers). By introducing this theoretical evaluation dimension, we aim to quantitatively compare the baseline hardware resource requirements of each algorithm at the mathematical mapping level. Specific statistics on parameter scale and computational load are shown in Table 4.

As shown in Table 4, traditional high-fidelity denoising networks typically rely on deep feature pyramids or global attention mechanisms to capture global time-frequency features, resulting in large model sizes. For instance, the PRISM model, which demonstrates a high denoising upper bound, possesses 70.42 M parameters and a theoretical computational overhead of 4.887 GFLOPs. Even relatively lightweight models, such as DnCNN or Transformer, maintain parameter counts in the range of 0.52 M to 0.55 M. Such computational overhead makes these models highly dependent on server-grade GPU memory, limiting their independent execution on memory- and power-constrained UAV edge terminals.

In contrast, the proposed SWIFT-Mamba exhibits a distinct lightweight advantage in terms of theoretical computational complexity. Attributed to the synergy between the Time-Frequency Dual-Track decoupling architecture and the 1D-SSM, the total parameter count of SWIFT-Mamba is merely 0.066 M, with a theoretical computational overhead of 0.147 GFLOPs. Compared with PRISM, SWIFT-Mamba reduces the parameter count by approximately three orders of magnitude and lowers FLOPs by about 33 times, while maintaining competitive reconstruction fidelity. This theoretical framework alleviates the burden of deploying resource-intensive networks on portable devices, providing a viable solution for implementing in situ verification systems in harsh environments.

#### 5.3.2. Single-Core Efficiency Evaluation Under Pure CPU Constraints

To evaluate the underlying hardware scheduling efficiency of SWIFT-Mamba on edge devices, this section investigates the model’s concurrent inference performance across varying numbers of threads (1, 2, 4, and 8) under pure CPU constraints. The CPU latency was evaluated on an Intel Core i5-1135G7 CPU @ 2.40 GHz with 16 GB RAM under Windows 11 64-bit. The software environment was Python 3.9.13, PyTorch 1.13.1, and SciPy 1.10.1. All CPU measurements were conducted using FP32 precision and batch size = 1 in the native PyTorch implementation. No deployment-specific acceleration, such as ONNX Runtime, TorchScript, operator fusion, or quantization, was applied. As shown in Table 5, the experimental results indicate that increasing the number of CPU threads does not yield the anticipated computational acceleration; instead, it exhibits a phenomenon of zero or even slight negative scaling. In single-thread mode, the model demonstrates its optimal operational efficiency, with a single-step inference latency of 348.31 ms. However, when computational resources are scaled to 8 threads, the inference latency rebounds to 355.91 ms, and the multi-core speedup ratio drops to 0.98.

The fundamental cause of this counterintuitive phenomenon lies in the highly lightweight architectural characteristics of the proposed model. Empirical data indicate that the parameter storage size of SWIFT-Mamba is approximately 0.25 MB, with only 0.066 M learnable parameters and 0.147 GFLOPs required for a single forward pass. Such a compact computational graph leads to an extremely small arithmetic workload per sample. Under this miniature workload, CPU inference latency is no longer dominated solely by floating-point operations. Instead, non-computational overheads, including operating-system-level thread scheduling, the wake-up of multiple idle cores, thread context switching, inter-core synchronization, memory access, and cache-coherence maintenance, become increasingly significant.

Consequently, increasing the number of CPU threads does not necessarily translate into a proportional reduction in inference latency. In the present implementation, the marginal acceleration introduced by intra-operator parallelism is largely offset, and in some cases even exceeded, by the additional overhead caused by multi-thread coordination. This explains why the measured latency remains nearly unchanged, or even slightly increases, when the number of CPU threads is enlarged. Therefore, the observed phenomenon is not contradictory to the low theoretical complexity of SWIFT-Mamba; rather, it reflects a typical deployment characteristic of ultra-lightweight neural networks under unoptimized CPU execution.

Overall, the multi-thread latency results further support the engineering value of SWIFT-Mamba, its advantage is not only reflected in a small parameter count and low FLOPs, but also in its compatibility with simple, low-power, and resource-constrained CPU execution. This makes SWIFT-Mamba a practical candidate for edge-oriented wavefront signal verification, where stable latency, low power consumption, and implementation simplicity are more valuable than aggressive multi-core acceleration.

### 5.4. Ablation Study

To systematically dissect the individual contributions of the architectural innovations and key hyperparameter configurations within the SWIFT-Mamba framework, we conducted extensive ablation studies on the laboratory-measured dataset. Crucially, to guarantee a rigorously fair comparison, all model variants were trained under strictly identical experimental conditions. As summarized in the configuration matrix in Table 6, these ablation variants were specifically designed to independently isolate and evaluate the impacts of STFT settings, phase-aware mask designs, bottleneck module placements, and dual-track architectures. The results of the corresponding evaluation metrics are shown in Table 7.

We first investigated the impact of time-frequency resolution by altering the STFT settings. The choice of the STFT window size governs the fundamental time-frequency resolution trade-off. We evaluated the STFT-512 variant, which enlarges the window size (*N_fft_*) from the default 128 to 512. As presented in Table 7, this alteration triggers a catastrophic performance collapse, with the SNR gain plummeting to 0.6584 dB and the SI-SDR severely degrading to −4.8856 dB. From a physical perspective, airborne wavefront vibration signals are characterized by highly dynamic, transient micro-fluctuations. A larger STFT window significantly smears the temporal resolution, preventing the network from accurately anchoring instantaneous phase shifts and abrupt transient impacts. This empirical evidence validates that a shorter STFT window (128) is an indispensable prerequisite for preserving the high-frequency temporal topology of dynamic wavefront signals.

Beyond time-frequency resolution, we verified the essentiality of phase-aware mask design in the complex domain. We replaced the Complex Ideal Ratio Mask (cIRM) with a real-valued Magnitude Ideal Ratio Mask (Mag-IRM), which exclusively enhances the amplitude spectrum while bypassing phase recovery. The results reveal a profound degradation: the Mag-IRM variant achieves an SI-SDR gain of only 4.4744 dB and a PCC of 85.54%, vastly inferior to the proposed model. In optical interferometry and wavefront sensing, the phase component encodes the critical spatial-temporal physical manifold of the vibration. Ignoring phase reconstruction inevitably leads to severe waveform structural distortion upon inverse transformation, underscoring the absolute necessity of the cIRM design for high-fidelity signal restoration.

Furthermore, the spatial placement of the State Space Model within the U-Net architecture significantly influences its representation learning efficiency. The Mamba-Shallow variant relocates the Physical Mamba blocks from the deep bottleneck to the shallow encoder layer. This premature sequence modeling results in a noticeable performance drop, with the SNR decreasing to 12.7213 dB. The underlying cause is that shallow feature maps retain massive, uncompressed spatial noise and local clutter, which severely distracts the Mamba scanning mechanism from capturing global temporal dependencies. Conversely, embedding the SSM at the deepest bottleneck—where local noise has been largely filtered by preceding convolutional strides—enables the model to focus computing resources entirely on highly abstracted, noise-free semantic manifolds, thereby maximizing its global receptive field efficiency.

To evaluate the efficacy of the time-frequency dual-track modeling, we investigated the individual contributions of the parallel paths. The Time-Only variant achieves a commendable SI-SDR of 14.7271 dB, outperforming the Freq-Only variant (11.5439 dB), which indicates that sequential temporal dynamics play a more dominant role in wavefront signal recovery than pure spectral features. However, the isolated Time-Only architecture’s SNR gain (15.3897 dB) remains significantly below that of the full SWIFT-Mamba model (19.6387 dB). This substantial performance gap quantitatively answers a critical concern: flattening a 2D time-frequency map into a 1D sequence for pure Mamba processing inherently disrupts the localized coupling features across adjacent frequency bins, leading to a significant loss of harmonic information (~4.26 dB in SNR). Without the parallel Freq-Scan path, the model struggles to explicitly anchor high-frequency harmonic distortions along the spectral axis. The remarkable performance leap of the full model unequivocally proves that the 1D-SSM (for long-range temporal lag) and the asymmetric large-kernel convolution (for local harmonic topology) are highly orthogonal and complementary.

Finally, we analyzed the impact of output constraint mechanisms. The Dual-Unb (unconstrained) variant suffers from potential numerical singularities during complex division, leading to sub-optimal metric stability. While Dual-Hard (Hard-Clipping) and Dual-Tanh (Tanh activation) provide necessary boundaries and improve the SNR to 17.5738 dB and 17.2853 dB, respectively, they suffer from inherent drawbacks. Hard-Clipping truncates physical transient peaks abruptly, causing zero-gradient regions, and Tanh is prone to saturation under extreme large-amplitude inputs. By replacing them with the proposed Soft-Clipping mechanism, the model achieves the highest SNR (19.6387 dB) and SI-SDR (16.1042 dB). This demonstrates that Soft-Clipping successfully maintains smooth gradient backpropagation and robust boundary protection, preventing network over-smoothing and phase fracturing under extreme working conditions.

In summary, the superior performance of SWIFT-Mamba is not merely an additive accumulation of modules, but the result of deeply synergistic, physics-inspired architectural choices tailored for airborne wavefront signal enhancement.

### 5.5. Interactive Web Demonstration

To facilitate independent validation and promote the practical application of the proposed SWIFT-Mamba algorithm, we have developed an interactive, web-based demonstration platform. While the core proprietary source code remains unexposed, this platform allows researchers and practitioners to seamlessly test the algorithm’s efficacy without requiring local computational resources or complex environment configurations.

The demonstration system is publicly accessible at: https://swift-mamba.streamlit.app.

Through this interface, external users can upload their own noisy 1D wavefront vibration data (in .npz or .csv formats) or choose from our database of randomly uploaded sample datasets. The serverless application processes the inputs in real-time and provides a direct visual comparison between the raw noisy signal and the SWIFT-Mamba denoised reconstruction. Please note that as this demonstration is deployed on a free community cloud platform, users may occasionally experience hardware-independent latency. Specifically, the system employs a “cold-start” resource management mechanism; after periods of inactivity, the application enters a sleep state to conserve resources. Initial access may require a brief delay to reallocate backend containers and reload the PyTorch environment and model weights, after which inference will return to real-time fluidity. Furthermore, the server operates under a strict 1 GB memory limit. Intensive platform traffic or the frequent uploading of excessively large data files may trigger memory threshold constraints, potentially resulting in temporary sluggishness or automatic application restarts. By providing this accessible tool, we aim to offer a transparent affirmation of the algorithm’s reproducibility, demonstrating its robustness and broad utility for real-world non-contact wavefront sensing applications. Please note that the online visualization results are intended for qualitative reference only. The definitive quantitative performance of the SWIFT-Mamba model is subject to the comprehensive offline experimental results detailed in Section 5.1 and Section 5.2 of this manuscript.

## 6. Conclusions

To address the strong nonlinear colored noise interference faced by airborne wavefront sensors in field environments, as well as the challenge of realizing “in situ verification” due to the constrained computational capacity of portable edge devices, this paper proposes SWIFT-Mamba, a lightweight time-frequency state-space model designed for edge computing. This model effectively balances the trade-off between the susceptibility of traditional digital filtering algorithms to high-frequency topological distortions and the substantial computational overhead of large-scale deep networks, providing a feasible engineering solution for the high-fidelity reconstruction and efficient denoising of wavefront signals. The main contributions and conclusions of this study are summarized as follows:

1. Proposed a Physics-Informed Dual-Track Decoupling Architecture: Rather than trivially integrating existing modules, a highly synergistic time-frequency dual-track perception mechanism was constructed. By orthogonally combining deep 1D-SSM with asymmetric large-kernel convolutions, the model captures global long-range sequential dependencies without disrupting local harmonic topologies. Comparative experiments demonstrate that, relative to the PRISM model (70.42 M parameters), SWIFT-Mamba significantly reduces the parameter count to 0.066 M and theoretical FLOPs to 0.147 G. This compact architecture effectively mitigates the computational burden associated with deploying deep networks on edge terminals.
2. Developed a Physical-Boundary Soft-Clipping Mechanism: To overcome the numerical singularities of traditional complex masking and the severe transient truncation caused by conventional activations (e.g., Hard-Clipping), a novel Soft-Clipping mechanism was applied. Ablation studies confirm that this mechanism guarantees smooth gradient backpropagation and strict boundary protection. When processing signals of varying frequencies (0.1–0.2 Hz), varying amplitudes (0.5–1.18 mm), and broadband fluctuations, the LSD of SWIFT-Mamba remains stable within the 0.08–0.09 dB range, demonstrating substantial capability in preserving high-frequency physical topology.
3. Validated Phase-Aware Complex Domain Robustness and Generalization: By successfully adapting the Mamba sequence modeling paradigm for complex domain optical interferometry via the Complex Ideal Ratio Mask (cIRM), the framework strictly preserves both magnitude and phase. Evaluated on a comprehensive dataset comprising over 60,000 samples, the model demonstrated consistent performance. On the synthetic dataset, its average SNR gain reached 19.82 dB, and the PCC achieved 98.55%. Furthermore, on the out-of-distribution laboratory-measured dataset, it maintained an SNR gain of 19.64 dB and an SI-SDR gain of 16.10 dB, proving its intrinsic capability to extract physical manifold features rather than merely overfitting to simulated noise distributions.

Despite the promising algorithmic outcomes, we objectively acknowledge the limitations of the current study as a foundational proof-of-concept. A prominent domain shift inherently exists between the semi-synthetic dataset evaluated herein and the authentic, non-stationary mixed noise distribution of true UAV field flights (e.g., highly coupled rotor vibrations and severe aerodynamic turbulence). Consequently, the absolute metric gains reported may present a certain degree of overestimation if directly extrapolated to extreme airborne scenarios. Furthermore, while this study successfully validates the algorithmic efficiency on CPU-constrained platforms (simulating edge-like thread scheduling limits), empirical deployment on true UAV-borne embedded hardware (e.g., Raspberry Pi, Rockchip NPUs) remains a future endeavor. It is worth noting that SWIFT-Mamba imposes no latent compatibility risks for hardware transplantation; its architecture relies exclusively on standard tensor operations without CUDA-dependent custom kernels, ensuring seamless export to edge formats like ONNX. Upon the acquisition of subsequent funding, future interdisciplinary research will prioritize hardware-software co-design. We plan to port the core SWIFT-Mamba algorithm to a high-efficiency edge computing platform integrated with an actual UAV payload, executing closed-loop field flight tests to systematically validate the system’s in situ verification capabilities under real-world aerodynamic complexities.

## Figures and Tables

**Table 1:** Baseline for the Overall Average Performance of the SWIFT-Mamba Model on Two Types of Datasets. The SNR, SDR, and SI-SDR values in the table are all gain values. The NMSE, PCC, and LSD values are all output values.

Average of the Evaluation Results						
Dataset Types	SNR (dB)	SDR (dB)	SI-SDR (dB)	NMSE	PCC (%)	LSD (dB)
Laboratory-measured signals	19.64	16.21	16.10	0.0292	98.78	0.076
Synthetic signals	19.82	15.54	15.42	0.0345	98.55	0.080

**Table 2:** Noise Reduction Performance Metrics for Different Input Amplitudes at the Same Frequency. The SNR, SDR, and SI-SDR values in the table are all gain values. The NMSE, PCC, and LSD values are all output values.

Evaluation Metrics in the Laboratory						
Amplitude (mm)	SNR (dB)	SDR (dB)	SI-SDR (dB)	NMSE	PCC (%)	LSD (dB)
0.50	17.47	14.56	14.37	0.0467	98.25	0.033
0.62	17.97	14.93	14.70	0.0412	98.43	0.029
0.75	17.79	14.89	14.73	0.0422	98.39	0.037
0.81	18.17	15.09	14.91	0.0396	98.48	0.035
0.87	19.02	15.94	15.81	0.0317	98.73	0.047
0.93	20.58	17.00	16.90	0.0223	99.01	0.057
1.00	20.66	16.97	16.89	0.0225	98.99	0.065
1.12	20.36	16.75	16.69	0.0233	98.96	0.087
1.18	19.99	16.60	16.63	0.0253	98.92	0.079

**Table 3:** Noise Reduction Quality Metrics for Different Target Frequencies at the Same Amplitude. The SNR, SDR, and SI-SDR values in the table are all gain values. The NMSE, PCC, and LSD values are all output values.

Evaluation Metrics in the Laboratory						
Frequency (Hz)	SNR (dB)	SDR (dB)	SI-SDR (dB)	NMSE	PCC (%)	LSD (dB)
0.10	20.44	16.82	16.76	0.0233	98.96	0.075
0.15	21.35	17.49	17.45	0.0186	99.11	0.095
0.18	21.05	17.10	17.08	0.0198	99.02	0.171
0.20	20.45	16.56	16.54	0.0226	98.90	0.176

**Table 4:** Lightweight computational metrics of different network models. Bold values indicate the best performance. The bold text refers to our methods and best results.

Model	Params(M)	FLOPs(G)
Vanilla U-Net	0.471	0.456
Attention U-Net	0.239	0.280
DCCRN	1.630	0.499
Conv-TasNet	0.989	0.176
DnCNN	0.521	2.119
Transformer	0.551	0.236
PRISM	70.419	4.887
**SWIFT-Mamba (Ours)**	**0.** **066**	**0.147**

**Table 5:** Performance evaluation of SWIFT-Mamba with varying numbers of threads under a pure CPU-constrained environment.

Threads	Params (M)	Model Size (MB)	Latency (ms)	Speedup Ratio
1	0.066	0.25	348.31	1.000
2	0.066	0.25	349.84	0.996
4	0.066	0.25	347.97	1.001
8	0.066	0.25	355.91	0.979

Note: CPU latency was measured with batch size = 1 using FP32 precision in the native PyTorch environment. The reported latency corresponds to an unoptimized single-sample software pipeline and includes STFT preprocessing, network inference, cIRM-based complex multiplication, and iSTFT reconstruction. File I/O, plotting, metric computation, and logging were excluded. Model size denotes parameter storage size rather than total runtime memory footprint.

**Table 6:** Configuration of SWIFT-Mamba ablation variants. The bold text refers to our methods.

Model Variant	STFT Size	Mask Design	Mamba Pos.	Time-Scan	Freq-Scan	Output Constraint
STFT-512	512	cIRM	Bottleneck	√	√	Soft-Clipping
Mag-IRM	128	Mag-IRM	Bottleneck	√	√	Soft-Clipping
Mamba-Shallow	128	cIRM	Shallow	√	√	Soft-Clipping
Time-Only	128	cIRM	Bottleneck	√	×	Soft-Clipping
Freq-Only	128	cIRM	Bottleneck	×	√	Soft-Clipping
Dual-Unb	128	cIRM	Bottleneck	√	√	None
Dual-Hard	128	cIRM	Bottleneck	√	√	Hard-Clipping
Dual-Tanh	128	cIRM	Bottleneck	√	√	Tanh
**Ours**	128	cIRM	Bottleneck	√	√	Soft-Clipping

Note: In the table, “√” indicates that the module is enabled in the corresponding model, while “×” indicates that the module is not used.

**Table 7:** Ablation results. The SNR and SI-SDR values in the table are all gain values. The PCC values are all output values. The bold text refers to our methods and best results.

Evaluation Metrics of Ablation Experiment (Laboratory)			
Model Variant	SNR (dB)	SI-SDR (dB)	PCC (%)
STFT-512	0.6584 ± 0.116	−4.8856 ± 1.337	50.11 ± 5.69
Mag-IRM	5.6795 ± 1.154	4.4744 ± 1.480	85.54 ± 3.88
Mamba-Shallow	12.7213 ± 1.136	12.7627 ± 1.147	97.43 ± 0.70
Time-Only	15.3897 ± 0.654	14.7271 ± 0.812	93.54 ± 0.6
Freq-Only	12.8575 ± 0.810	11.5439 ± 0.937	91.15 ± 1.4
Dual-Unb	16.8422 ± 0.505	14.7402 ± 0.959	95.53 ± 0.5
Dual-Hard	17.5738 ± 0.406	15.7190 ± 0.958	97.02 ± 0.4
Dual-Tanh	17.2853 ± 0.598	15.4279 ± 0.986	96.25 ± 0.6
**Ours**	**19.6387 ± 0.306**	**16.1042 ± 0.853**	**98** **.** **78 ± 0** **.** **3**

## Data Availability

The data presented in this study are not publicly available due to ongoing future research utilizing this dataset. The data are available on reasonable request from the corresponding author.

## Abbreviations

The following abbreviations are used in this manuscript:

1D-SSM	One-Dimensional State Space Model
cIRM	Complex Ideal Ratio Mask
EMD	Empirical Mode Decomposition
FLOPs	Floating Point Operations
LSD	Log-Spectral Distance
LSTM	Long Short-Term Memory
NMSE	Normalized Mean Square Error
PCC	Pearson Correlation Coefficient
SDR	Signal-to-Distortion Ratio
SiLU	Sigmoid Linear Unit
SI-SDR	Scale-Invariant Signal-to-Distortion Ratio
SNR	Signal-to-Noise Ratio
SSM	State Space Model
STFT	Short-Time Fourier Transform
iSTFT	Inverse Short-Time Fourier Transform
